# Systematic Review of Outcome Measures in Pharmacologically Managed Chronic Pain: Informing a New Outcome Framework for Healthcare Provider‐Led Pharmacotherapy Services

**DOI:** 10.1111/jep.70029

**Published:** 2025-02-26

**Authors:** Abdulrahman Sharaf, Emma Dunlop, Natalie Weir, Rosemary Newham, Sumaya Alsalah, Marion Bennie

**Affiliations:** ^1^ Strathclyde Institute of Pharmacy and Biomedical Sciences University of Strathclyde Glasgow UK; ^2^ Department of Clinical Pharmacy Salmaniya Medical Complex, Government Hospitals Manama Bahrain; ^3^ Department of Pharmacy Primary Healthcare Centers Manama Bahrain

**Keywords:** chronic pain, medicines, outcome measurement, outcomes, pharmacological, pharmacy, systematic review

## Abstract

**Background and Objective:**

Chronic pain represents a global burden, highlighting the necessity for accurate outcome measures in treatment evaluation. This systematic review aims to identify what outcome measures and tools are applied in chronic pain primary care‐based pharmacotherapy services.

**Databases and Data Treatment:**

The MEDLINE, Embase, and CINAHL databases, along with the reference lists of published articles, were systematically searched from 2013 to July 2023. This search included observational studies that employed pharmacological interventions recommended by the World Health Organisation pain ladder and the Scottish Intercollegiate Guidelines Network guidelines. The studies targeted chronic pain patients treated in outpatient settings and examined five predefined outcomes: health‐related quality of life (HRQoL), cost‐effectiveness, medication optimisation, adverse events, and patient experience. The quality of included studies was assessed using the Newcastle‐Ottawa Scale (NOS).

**Results:**

Among the 23 studies included a total of 51 outcome measurement tools were employed to assess the five predefined outcomes, involving 44,472 patients with chronic pain. Fifteen were cohort studies, while 8 were cross‐sectional surveys or questionnaire‐based. Most studies focused on one to two outcomes only (*n* = 19; 82.6%). HRQoL emerged as the primary outcome studied across all 23 studies (100%), predominantly assessed through the Brief Pain Inventory (BPI) tool (*n* = 9; 39.1%). Conversely, the least studied outcomes were medication optimisation and cost‐effectiveness. The timing of measurement post‐intervention and follow‐up durations displayed significant variability across the studies.

**Conclusions:**

This review identifies gaps in enabling a more holistic assessment of pharmacotherapy services and underscores the need for enhanced consistency via standardised tools in clinical practice.

## 1 Introduction

Chronic pain is defined as nonmalignant pain lasting more than 3 months, including: primary chronic pain not attributed to other conditions; or secondary chronic pain resulting from an underlying disease or condition [1, 2]. Chronic pain is a complex debilitating condition affecting millions of individuals worldwide [3]. The treatment of chronic pain frequently involves the use of various medicines, either as monotherapy or in combination, including paracetamol, opioids, nonsteroidal anti‐inflammatory drugs (NSAIDs), antidepressants, and anticonvulsants [4]. It was reported that the medication‐based dimension of chronic pain treatment was used in 85% of patients with chronic pain, while 25% reported incorporating nonpharmacological treatments into their treatment approach [5]. This underscores the need for assessment tools focused on medication use within routine care. Such tools should offer comprehensive evaluation across various domains while remaining practical and feasible beyond the confines of clinical trials. Moreover, whilst clinical trials provide valuable insights into the efficacy of pain‐management interventions, understanding how the measures perform in real‐world settings is essential for ensuring their relevance, reliability and validity in everyday practice.

There is significant variation in the use of outcome measures in chronic pain management by healthcare providers and researchers [6]. Additionally, existing evidence identifying these measures may lack inclusivity, due to being limited to specific types of chronic pain and targeting particular methods (e.g., patient self‐report), and not encompassing all possible outcomes [6, 7, 8]. In a published article on development of an outcome framework for pharmacotherapy and disease management services in Scotland, consensus involving pharmacy professionals was reached on five key outcome areas for the framework: patient experience; medication‐related adverse events; cost‐effectiveness; medication optimisation; and health‐related quality of life (HRQoL) [9]. In Scotland, the importance of identifying useful pain outcomes measures is exemplified by the expanding role of pharmacists, who have recently integrated within general practice. The Scottish Government funded the recruitment of full time General Practice Clinical Pharmacists as part of plans to alleviate pressures in primary care. Their role in general practice includes the delivery of a ‘Pharmacotherapy Service’, where they may offer pharmacy‐led pain clinics [10]. The identification of appropriate outcome measures for use in such pharmacist‐led clinics within general practice will help evaluate the effectiveness of the Pharmacotherapy Service in Scotland [10] and will support evidence‐based service delivery within Scotland's primary care sector.

To identify suitable outcomes measures for potential use in pharmacy‐led pharmacotherapy services, such as the Scottish Pharmacotherapy Service, the aim of this systematic review was to identify and evaluate outcome measures and associated tools applied in primary care‐based pharmacotherapy pain management services provided by healthcare professionals.

## 2 Methods

A systematic review can be defined as attempting to collate empirical evidence from a relatively smaller number of studies pertaining to a focused research question [11]. Therefore, given the scope of this study, a systematic review methodology was conducted and reported following the Preferred Reporting Items for Systematic Reviews and Meta‐Analyses (PRISMA) guidelines [12]. The review was registered in PROSPERO with the following ID (CRD42023442201).

### 2.1 Search Strategy and Data Sources

A search was conducted after consultation with a reference librarian, and searches were performed in the CINAHL, EMBASE, and MEDLINE databases from 2013 to the present (July 2023). The search strategy combined MeSH terms and keywords related to: chronic pain; analgesics; opioids; outpatient; and primary care. Additionally, a manual search of the reference lists of retrieved journal articles, including systematic reviews, was performed to identify any additional relevant records that met the inclusion criteria. The complete search strategy can be found in the Supporting Information Tables S1 and S2.

### 2.2 Inclusion Criteria

The included studies in this systematic review met the following criteria: observational studies were selected to reflect real‐world clinical practice and included participants aged 18 years and above with non‐cancer‐related chronic pain, who were receiving treatment and follow‐up in outpatient settings. Chronic pain was defined as per the International Association for the Study of Pain (IASP) [1] and the National Institute for Health and Care Excellence (NICE) guidelines NG193 [2]. Studies should be using measures to assess the 5 predefined outcomes in chronic pain management services. The interventions encompassed treatments recommended by the World Health Organisation (WHO) pain ladder [13] and the Scottish Intercollegiate Guidelines Network (SIGN) guidelines [14], including: paracetamol; NSAIDs; opioids; antidepressants; anticonvulsants; and topical treatments such as capsaicin, rubefacients, and lidocaine. Pharmacological treatments were included given the study's focus on predefined criteria related to pharmacotherapy and the Scottish pharmacotherapy service and outcomes framework. Exclusions from this review included non‐English language studies, clinical trials, case reports, case series, and narrative reviews. All studies examining cancer‐related chronic pain or acute pain were also excluded. Interventions solely involving non‐pharmacologic treatments (e.g., cognitive‐behavioural therapy, etc.) were excluded as well.

### 2.3 Study Selection

The study selection process was conducted using the Rayyan© software [15]. After eliminating any duplicated records, two reviewers (A.S. and S.A.) independently performed title and abstract screening, and screening of full texts of potentially eligible studies. Excluded studies during full‐text screening were documented, with a justification provided for their exclusion. In cases where discrepancies arose, a third reviewer (N.W. or E.D.) were involved to resolve disagreements.

### 2.4 Data Extraction

Reviewer A.S. independently extracted the relevant data from the included studies and was validated by the second reviewer, S.A, using a pilot‐tested data extraction Microsoft Excel© sheet (see Supporting Information Tables [Link], [Link], [Link]). Table S3 outlines the broader context of the studies, including their aims and key findings; Table S4 focuses on the populations studied, types of pain assessed, and therapies included; and Table S5 highlights the outcome measurement tools, which are central to our study's purpose. Throughout the data extraction process, any discrepancies or uncertainties that emerged were resolved through discussions and consensus with the authors, N.W. and E.D.

The data extracted from the included studies encompassed baseline study characteristics, including: authors; publication year; sample size; study aim; characteristics of the study population; description of the intervention and comparison; assessed outcomes; key findings; study groups; setting; type of pain; medication administered; and involvement of healthcare professional. Additionally, details on the outcome measures from the studies were extracted, which included: information on the measurement tools employed; performer of the outcome assessment (i.e. patient or specific healthcare professional); if the tool was validated; and frequency of tool use. All measurement tools were extracted from the included studies and classified according to the five predefined outcomes. Working definitions for each outcome were developed based on a comprehensive search in recently published studies and health organisations [16, 17, 18, 19, 20, 21, 22] to assist in the clarification of the outcomes. The definitions of the predefined outcomes are outlined in Table 1. During data extraction, relevant outcome measures were categorised into domains depending on the areas they covered. Where possible, this process utilised existing domains, such as the World Health Organisation HRQoL domains and subdomains [24].

**Table 1:** Definition of the outcomes.

Outcomes [9]	Working definition
Health‐related quality of life	Value is assigned to the duration of life, as modified by impairments, functional states, perceptions, and social opportunities influenced by disease, injury, treatment, or policy. This concept encompasses physical health (pain, sleep), psychological health (positive and negative feelings), level of independence (mobility and activity), and social relationships (personal and social relationships) [23, 24].
Patient experience	Patient‐reported perception of a healthcare organisation and their journey across the continuum of care encompasses aspects such as communication, access to care, coordination of care, respect and dignity, involvement in care, physical comfort, and overall satisfaction [17, 18, 20, 22].
Medication related adverse events	Negative outcomes or harmful effects that occur as a result of medication use, which could include side effects, medication errors, and any unintended consequences of medication use [25].
Medication optimisation	The systematic and comprehensive process involves maximising the benefits of medication use while minimising potential risks and adverse effects. This may involve medication additions, changes in medication intensity, discontinuation, treatment restart after discontinuation, and ensuring medication appropriateness [21, 26].
Cost‐effectiveness	Assessing the value or efficiency of a healthcare intervention or programme involves comparing the costs incurred with the outcomes achieved. This can be done using measures such as the Incremental Cost‐Effectiveness Ratio (ICER) or the Incremental Cost‐Utility Ratio (ICUR) [19, 21, 27, 28].

### 2.5 Risk of Bias and Quality Assessment

The methodological quality of each study was evaluated by two independent reviewers, A.S. and S.A., with any discrepancies resolved with the involvement of the reviewers N.W. and E.D. The Newcastle‐Ottawa Scale (NOS) was utilised to assess the methodological quality of the observational studies [29].

## 3 Results

**Figure 1:**
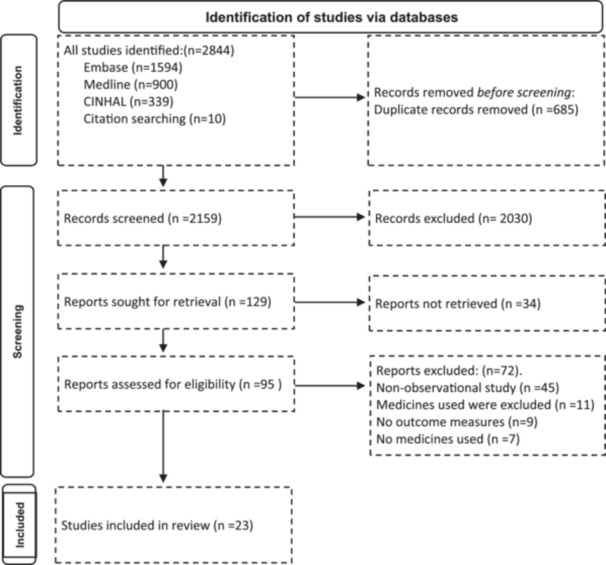
PRISMA flow diagram of the search strategy and results.

Figure 1 displays the PRISMA flow diagram of the search strategy and results. In total, 2,833 articles were identified through database searching. After removing all duplicates, 2,148 abstracts were screened for eligibility according to the predefined inclusion and exclusion criteria. In addition, 10 articles were identified by a manual search of the reference lists of retrieved journal articles. After further exclusions, 23 studies were included in the review.

### 3.1 Study Characteristics

Among the 23 included studies, 65.2% (*n* = 15) were cohort studies, while 34.7% (*n* = 8) were cross‐sectional surveys or questionnaire‐based studies. These studies involved a total of 44,472 patients, with a mean age ranging from 44 to 70 years, and males comprising a range 23%–71% of the total patient population.

In total, 51 outcome measures were used in the 23 reviewed studies. The studies examined various chronic pain conditions, with a majority focusing on mixed type of pain (*n* = 14/23; 60.8%), followed by musculoskeletal pain (*n* = 6/23; 26%), and a neuropathic pain (*n* = 3/23; 13%). Additionally, among the five predefined outcomes, 39.1% of the studies (*n* = 9/23) focused on assessing a single outcome, followed by 43.4% of studies (*n* = 10/23) assessing two outcomes. Furthermore, 13% (*n* = 3/23) of the studies investigated three outcomes, while only one study (4.3%) was the most comprehensive and assessed four outcomes. Treatment approaches varied among the studies, with monotherapy being used in 9 studies (39.1%), and a combination therapy approach described in 14 studies (60.8%). The most frequently investigated drugs were opioids, which were utilised in 19 studies (82.6%), followed by NSAIDs in 12 studies (52.1%).

Among the included studies, 21 (91.3%) focused on treating ongoing chronic pain, while two studies (8.6%) aimed to deprescribe treatment.

HRQoL was the main studied outcome in all 23 studies (100%). Patient experience outcomes were assessed in 7 studies (30.4%), medication‐related adverse events in 6 studies (26%), and the least studied outcomes were medication optimisation and cost‐effectiveness, each assessed in only three studies (13%). Additionally, the majority of tools identified were used to assess HRQoL outcomes (33 out of 51 tools; 64.7%), followed by tools used to assess patient experience (10 out of 51 tools; 19.6%).

Thirteen studies (56.5%) were conducted in primary care settings, 9 (39.1%) were conducted in hospital‐based outpatient clinics, and one (4.3%) was conducted in a rehabilitation centre. These studies were conducted in 9 countries, including the USA (*n* = 9; 39.1%); Spain (*n* = 4; 17.4%); Canada (*n* = 3; 13.0%); and Japan (*n* = 2; 8.7%). One study (*n* = 1; 4.3%) was conducted in each of Brazil, Germany, India, and the UK. Table 2 presents the baseline characteristics of the included studies.

**Table 2:** Baseline characteristics of included studies (*n* = 23).

Author & Year, Country	Study design	Population characteristics	Pain condition	Treatment used	Outcomes assessed
Nadkarni et al., [30] India	Cohort	**Sample size (*n*):** 100 **Mean (SD) age:** 44.68 (12.07) **Male (*n* (%)):** 69 (69) **Setting:** Pain Specialty Outpatient at Hospital	Neuropathic pain	NSAIDs^a^ Opioids TCAs^a^ Anticonvulsants	Pain improvement Tolerability
Robinson et al., [31] USA	Cross‐Sectional	**Sample size (*n*):** 572 **Mean (SD) age:** 64.9 (11.3) **Male (*n* (%)):** 226 (39.5%) **Setting:** Primary care	Osteoarthritis	Paracetamol NSAIDs^a^ Duloxetine Opioids	Patient Satisfaction with medication Patient expectation of effectiveness of medication
Zinboonyahgoon et al., [32] Thailand	Cohort	**Sample size (*n*):** 29 **Age (Median (range)):** 47.4 (15–79) **Male (*n* (%)):** 16 (54) **Setting:** Pain clinic at Hospital	Chronic refractory pain	Conventional management (CMM)	Pain reduction Improvement of function Quality of life Cost effectiveness
Moreira de Barros et al., [33] Brazil	Cohort	**Sample size (*n*):** 262 **Mean (SD) age:** 59.2 (14.9) **Male (*n* (%)):** 98 (37.3) **Setting:** Outpatient specialised Pain Management at Hospital	Chronic pain (Nociceptive, neuropathic and mixed type)	Methadone Morphine	Analgesic effect Side effects Pain intensity
Ganguly et al., [34] USA	Cross‐Sectional	**Sample size (*n*): 166** **Mean (SD) age:** — **Male (*n* (%)):** 108 (65) **Setting:** HIV primary care	Pain in HIV patients	Opioid	Satisfaction with pain management Patient barriers to pain management
Gudin et al., [35] USA	Cross‐Sectional	**Sample size (n):** 199 **Mean age:** 46 **Male (*n* (%)):** 72 (36) **Setting:** Outpatient clinic	Arthritis Neuropathy/radiculopathy Myofascial/musculoskeletal	OTC^a^ and prescribed NSAID^a^ Opioid Anticonvulsant and muscle relaxant	Pain intensity and interference Patient satisfaction Changes in concurrent pain medications Side effect
Kaboré et al., [36] Canada	Cohort	**Sample size (*n*):** 160 **Mean (SD) age:** 51.3 (12.5) **Male (*n* (%)):** 49 (42.2) **Setting:** Pain clinic	Chronic non‐cancer pain	Opioid	Pain severity Quality of life
Ramírez‐Maestre et al., [37] Spain	Cross‐Sectional	**Sample size (*n*):** 675 **Mean (SD) age:** 45.4 (12.9) **Male (*n* (%)):** 274 (41) **Setting:** Primary care centres	Chronic back pain	Opioids Other analgesics	Pain intensity Depressive symptoms Pain catastrophising Pain acceptance
Sicras‐Mainar et al., [38] Spain	Cohort	**Sample size (*n*):** 38,539 **Mean (SD) age:** 70.8 (14.3) **Male (*n* (%)):** 10,791 (28) **Setting:** Primary care centres	Osteoarthritis	Opioids Other analgesics	Health/non‐health resource utilisation Costs Treatment adherence Pain change Cognitive functioning Dependence
Lee et al., [39] USA	Cross‐Sectional	**Sample size (*n*):** 178 **Mean (SD) age:** — **Male (*n* (%)):** — **Setting:** A&M Health Science Centre Opioid Task Force	Chronic non‐cancer pain	Narcotics Non‐narcotics Opioids	Patient satisfaction with pain care Patient satisfaction with pain relief Perceptions about participation in treatment decisions Confidence in physicians
Taguchi et al., [40] Japan	Cohort	**Sample size (*n*):** 360 **Mean (SD) age:** Group 1: 58.3 (15.9) Group 2: 66.4 (15.8) **Male (*n* (%)):** Group 1: 77 (53.1) Group 2: 68 (30.4) **Setting:** Primary care	Chronic cervical radiculopathy with upper limb radiating pain	Pregabalin Anticonvulsant Antidepressant Opioid Non‐opioid Analgesics	Pain related sleep interference scale Pain numerical rating scale Neck disability index Quality of life
Wayne et al., [41] USA	Cohort	**Sample size (*n*):** 309 **Mean (SD) age:** Group 1: 50.18 (16.65) Group 2: 52.08 (15.88) **Male (*n* (%)):** — Group 1: 42 (31.3) Group 2: 38 (26.4) **Setting:** Clinical Centre at hospital	Chronic back pain	Integrative or conventional care	Quality‐adjusted life years (QALYs) Incremental cost‐effectiveness ratio (ICER)
McCann et al., [42] USA	Cohort	**Sample size (*n*):** 29 **Mean age:** 66.9 **Male (*n* (%)):** 20 (69) **Setting:** Rural Primary Care Office	Nonmalignant chronic pain (neck, upper and lower back pain, shoulder, knee, polyarthralgia, peripheral neuropathy)	Opioid	Whether patients elected to remain on opioids, wean opioids, or transfer care Evaluation of pain Functional status Mood
Elsesser, Cegla [43] Germany	Cross‐Sectional	**Sample size (*n*):** 333 **Mean (SD) age:** Group 1: 62.74 (15.34) Group 2: 65.86 (13.48) **Male (*n* (%)):** Group 1: 24% Group 2: 26.5% **Setting:** Clinic for Pain Medicine in primary care	Non‐cancer chronic pain including neuropathic pain	Opioids Analgesics Coanalgesics	Pain Functional disability Psychological wellbeing Quality of life
Ghodke et al., [44] USA	Cohort	**Sample size (*n*):** 171 **Mean (SD) age:** Group 1: 59.6 (10.32) Group 2: 59.9 (8.83) **Male (*n* (%)):** 54 (31.5) **Setting:** Internal Medicine Pain Service	Moderate to severe osteoarthritis	Opioid	Average pain scores Lowest pain scores Morphine equivalence
Vogler et al., [45] USA	Cohort	**Sample size (*n*):** 35 **Mean (SD) age:** 58 (11) **Male (%):** 23% **Setting:** Primary care clinic	Chronic non‐cancer pain (Back pain, upper and lower extremity pain, neck pain)	Opioid	Functional Status Knowledge Behaviour Satisfaction
White et al., [46] Canada	Cross‐Sectional	**Sample size (*n*):** 102 **Mean (SD) age:** 45.9 (11.7) **Male (*n* (%)):** 57 (55.9) **Setting:** Rehabilitation centre	Myofascial pain Other musculoskeletal pain Neuropathic pain, Mixed pain, Psychological diagnoses (pain disorder) Complex regional pain syndrome	Opioid	Pain interference Pain severity Depression Opioid intake
Igarashi et al., [47] Japan	Cohort Study	**Sample size (*n*):** 331 **Mean age (Mean (SD)):** 60 **Male (*n* (%)):** — **Setting:** Primary care clinic	Chronic low back pain with accompanying neuropathic pain	Pregabalin	Quality‐adjusted life years (QALYs) Direct medical costs Hospitalisation costs Productivity losses
Dunn et al., [48] USA	Cross‐Sectional Study	**Sample size (*n*):** 227 **Mean age (Mean (SEM)):** 46.00 (0.87) **Male (*n* (%)):** 64 (47) **Setting:** Addiction treatment clinic	Upper and lower extremities back, head, neck, shoulders, chest, abdomen and hips pain	Opioid Ibuprofen Gabapentin	Pain intensity
Jouini et al., [49] Canada	Cohort Study	**Sample size (*n*):** 486 **Mean age (Mean (SD)):** 58.4 (12.5) **Male (*n* (%)):** 157 (32.3) **Setting:** Primary care clinic	Osteoarthritis and other osteopathologie Chronic back pain Chronic neck pain Fibromyalgia Tendinitis, bursitis, capsulitis, epicondylitis	Paracetamol NSAIDs^a^ Anticonvulsants Antidepressants Muscle relaxants Opioids	Pain intensity Emotional wellbeing Satisfaction with pain treatment Barriers/beliefs/attitudes about pain and its treatment
Ashworth et al., [50] UK	Cohort Study	**Sample size (*n*):** 715 **Mean age (Mean (SD)):** Group 1: 45.77 (9.87) Group 2: 46.27 (9.36) Group 3: 44.07 (10.69) Group 4: 47.64 (9.39) **Male (*n* (%)):** Group 1: 44% Group 2: 33% Group 3: 27% Group 4: 24% **Setting:** UK primary care	Chronic lower back pain	Opioid	Disability
Pérez et al., [51] Spain	Cohort Study	**Sample size (*n*):** 1,845 **Mean age (Mean (SD)):** Group 1: 58.6 (12.5) Group 2: 59.7 (13.0) Group 3: 60.4 (12.4) **Male (*n* (%)):** Group 1: 289 (39.6) Group 2: 307 (42.2) Group 3: 84 (44.7) **Setting:** Primary care clinic	Neuropathic pain	Pregabalin Analgesics	Pain intensity Disability Sleep disturbances Anxiety and depression Quality of life Health care resource utilisation Corresponding costs
Blanco Tarrio et al., [52] Spain	Cohort Study	**Sample size (*n*):** 1,670 **Mean age (Mean (SD)):** 58.5 (13.7) **Male (*n* (%)):** 689 (41) **Setting:** Spanish primary care	Neuropathic pain	Pregabalin Anticonvulsant Antidepressant Opioid Non‐opioid analgesics	Pain Intensity Interference of pain with activities Number of days with no or mild pain Treatment satisfaction

^a^ Abbreviations: NSAIDs ‐ Nonsteroidal anti‐inflammatory drugs; OTC ‐ Over the counter; TCAs ‐ Tricyclic antidepressants;

#### 3.1.1 Quality Assessment

Table 3 presents the quality assessment results for the included studies using the NOS approach. Among the 15 cohort studies, 13 were classified as high quality, and three studies were rated as moderate quality. For the 8 cross‐sectional studies, 7 achieved high quality, while one study was deemed of moderate quality.

**Table 3:** Summary result of risk of bias and Quality assessment.

Study ID (ref)	Selection (Max 4 stars)	Comparability (Max 2 stars)	Outcome (Max 3 stars)	Score out of 9 (Quality rating)^a^
**Modified NOS for cross‐sectional studies**				
Robinson et al., [31]	***	*	***	7 (High)
Ganguly et al., [34]	**	**	***	7 (High)
Gudin et al., [35]	***	*	***	7 (High)
Ramírez‐Maestre et al., [37]	****	**	***	9 (High)
Lee et al., [39]	***	**	***	8 (High)
Elsesser, Cegla [43]	***	**	***	8 (High)
White et al., [46]	**	**	**	6 (Moderate)
Dunn et al., [48]	***	**	***	8 (High)
**NOS for cohort studies**				
Nadkarni et al., [30]	****	**	**	8 (High)
Zinboonyahgoon et al., [32]	***	*	***	7 (High)
Moreira de Barros et al., [33]	**	**	***	7 (High)
Kaboré et al., [36]	***	**	**	7 (High)
Sicras‐Mainar et al., [38]	***	**	***	8 (High)
Taguchi et al., [40]	***	**	***	8 (High)
Wayne et al., [41]	***	**	**	7 (High)
McCann et al., [42]	***	**	**	7 (High)
Ghodke et al., [44]	***	*	**	6 (Moderate)
Vogler et al., [45]	**	—	***	5 (Moderate)
Igarashi et al., [47]	***	**	***	8 (High)
Jouini et al., [49]	**	*	**	5 (Moderate)
Ashworth et al., [50]	***	**	**	7 (High)
Pérez et al., [51]	***	**	***	8 (High)
Blanco Tarrio et al., [52]	***	**	***	8 (High)

^a^ The quality assessment scores range from 0 to 9 stars, where 7 or more stars indicate high quality, 4 to 6 stars indicate moderate quality, and 1 to 3 stars indicate low quality.

### 3.2 Analysis of the Outcome Measures

**Figure 2.**
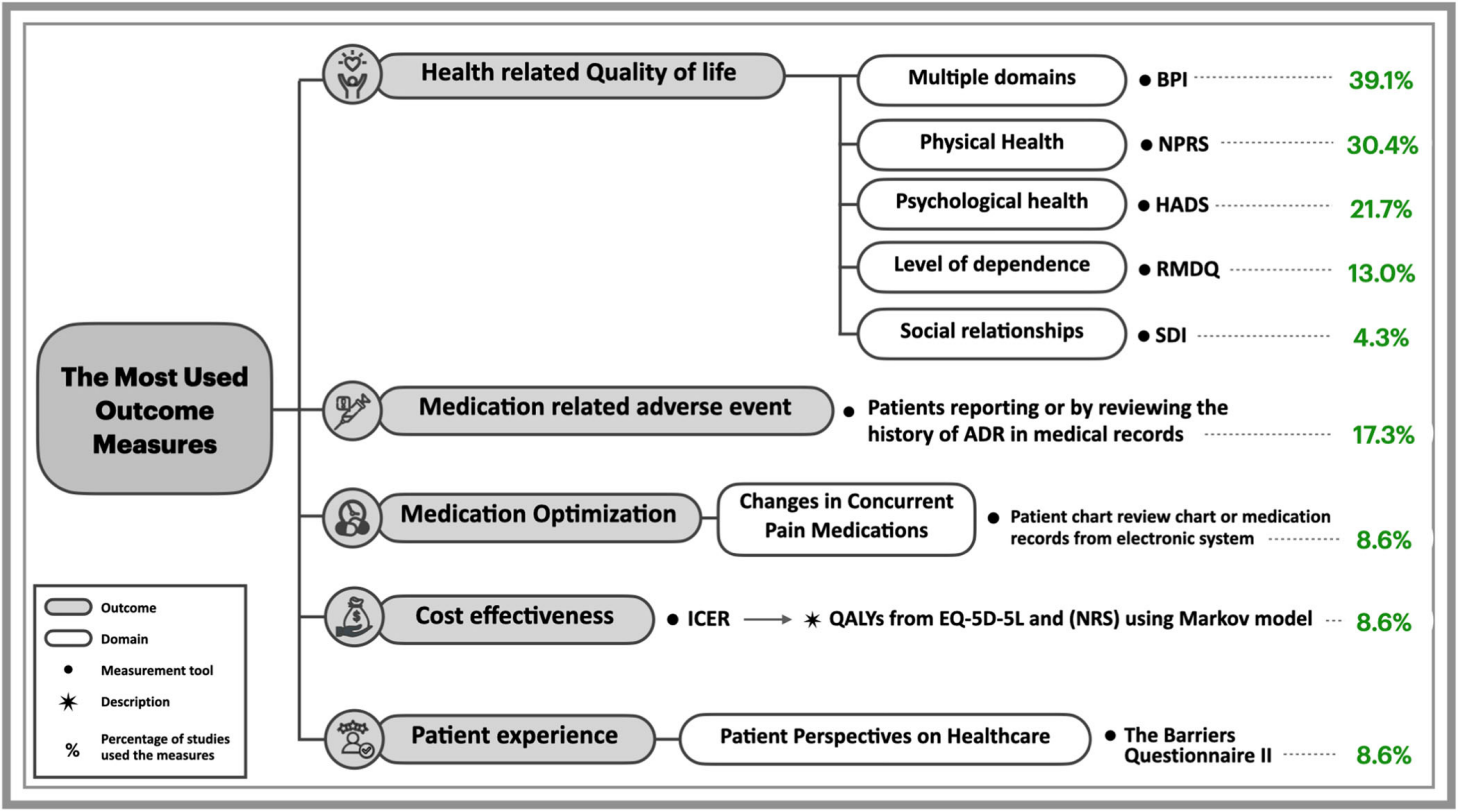
The most used outcome measures across all domains (*n =* 23) ADR, Adverse Drug Reaction; BPI, The Brief Pain Inventory; EQ‐5D‐5L, The EuroQol 5 Dimension 5 Level; HADS, Hospital Anxiety and Depression Scale; ICER, Incremental cost‐effectiveness ratio; NPRS, Numerical Pain Rating Scale; NRS, Numerical Rating Scale; QALYs, Quality‐Adjusted Life Years; RMDQ, Roland‐Morris Disability Questionnaire; SDI, Sheehan Disability Inventory.

The tools used were inadequately described (e.g., frequency of use, reasons for selection) in 60.8% of the studies (*n* = 14/23). A measurement tool was considered validated if the study confirmed the validation of the tool in their study process, regardless of its quality and the disease entity studied. Tables 4, 5, 6, 7, 8 presents the identified outcome measures and their usage frequencies in the included studies. Additionally, Figure 2 displays the most utilised tool across all identified domains.

**Table 4:** HRQoL outcome measure tools identified and their usage frequencies in the included studies (*n* = 23).

Domains	Subdomain (s)	Tools	*N* (%)
Physical health	Pain intensity	Numerical pain rating scale (NPRS) [31, 38, 43, 46, 47, 49, 50]	7 (30.4)
		Short‐Form McGill Pain Questionnaire [30, 51]	2 (8.6)
		11‐point numeric verbal scale [33]	1 (4.3)
	Pain intensity (neuropathic pain)	Neuropathic 4 Pain Questions (DN4) [36, 52]	2 (8.6)
		PainDETECT questionnaire [43]	1 (4.3)
	Sleep and rest	Pain‐Related Sleep‐Interference Scale (PRSIS) [40, 47]	2 (8.6)
		Medical Outcomes Study Sleep Scale (MOS‐Sleep) [51]	1 (4.3)
		Chronic Pain Sleep Inventory [49]	1 (4.3)
Psychological health	Cognitive and emotional response to pain	Hospital Anxiety and Depression Scale (HADS) [37, 43, 49, 50, 51]	5 (21.7)
		Beck Depression Inventory‐I (BDI)[36, 46]	2 (8.6)
		Pain Catastrophising Scale (PCS) [36, 37]	2 (8.6)
		Zung Depression scale [42]	1 (4.3)
		Tampa Scale of Kinesiophobia [50]	1 (4.3)
		Mini‐Mental State Examination (MMSE) [38]	1 (4.3)
		Chronic Pain Acceptance Questionnaire (CPAQ‐SV) [37]	1 (4.3)
Level of dependence	Performance status and disability	Karnofsky Performance Status [33]	1 (4.3)
		The modified Pain Disability Index (mPDI) [43]	1 (4.3)
		Pain Self‐Efficacy Questionnaire [50]	1 (4.3)
		Physical Function subscale [31]	1 (4.3)
		Barthel index [38]	1 (4.3)
	Back pain‐related functional limitation	Roland‐Morris Disability Questionnaire [RMDQ] [41, 42, 50]	3 (13.0)
		Oswestry Disability Index Survey [45]	1 (4.3)
	Neck pain‐related functional limitation	Neck Disability Index (NDI) [40]	1 (4.3)
Social relationships	Social life and responsibilities	Sheehan Disability Inventory (SDI) [51]	1 (4.3)
Tools assessing multiple domains of HRQoL	Assess pain intensity and impact on daily activities	The Brief Pain Inventory (BPI) [34, 35, 36, 42, 44, 46, 48, 49, 51]	9 (39.1)
	Mobility, self‐care, usual activities, pain/discomfort, and anxiety/depression	European Quality of Life‐5 Dimensions (EQ‐5D) [31, 32, 40, 47, 51]	5 (21.7)
	Physical and mental health functioning	Short Form‐12 Health Survey (SF‐12v2) [36, 41]	2 (8.6)
	Pain intensity, enjoyment of life, and general activity	Pain Intensity, Enjoyment of life, and General Activity (PEG) score [36, 45]	2 (8.6)
	Physical health, psychological health, social relationships, and environment	World Health Organisation Quality of Life‐ Brief Version (WHOQoL‐BREF) [43]	1 (4.3)
	Pain intensity, impact on daily activities, mood, and sleep	German Pain Questionnaire (GPQ) [43]	1 (4.3)
	Pain intensity and disability	Chronic Pain Grade Scale (CPG) [43]	1 (4.3)
	Pain intensity, physical function, and emotional wellbeing	American Pain Society Patient Outcome Questionnaire [39]	1 (4.3)
	Health‐related habits and behaviours	Marburg Questionnaire on Habitual Health (MQHH) [43]	1 (4.3)

**Table 5:** Patient Experience outcome measure tools identified and their usage frequencies in the included studies (*n* = 23).

Domains	Tools	*N* (%)
Patient perspectives on healthcare: experience, satisfaction, knowledge, and health status change	The Barriers Questionnaire II [34, 49]	2 (8.6)
	Patient Satisfaction Survey [45]	1 (4.3)
	Satisfaction ratings scale [35]	1 (4.3)
	Treatment Satisfaction for Medication Questionnaire (SATMED‐Q) [51]	1 (4.3)
	American Pain Society Patient Outcome [39]	1 (4.3)
	Patient satisfaction with pain management [34]	1 (4.3)
	Pre‐ and post‐knowledge assessment question [45]	1 (4.3)
	The Pain Treatment Satisfaction Scale (PTSS) [49]	1 (4.3)
	The Patient Global Impression of Change (PGIC) [40]	1 (4.3)
	5‐point Likert scale [31]	1 (4.3)

**Table 6:** Medication related adverse event outcome measure tools identified and their usage frequencies in the included studies (*n* = 23).

Domains	Tools	*N* (%)
Patient‐reported side effects and tolerability	Patients reporting or by reviewing the history of drug‐related adverse events in medical records [30, 33, 35, 40]	4 (17.3)
	Patient self‐administered questionnaire [49]	1 (4.3)
Risk assessment for adverse events	Screener and Opioid Assessment for Patients with Pain (SOAPP‐R) risk assessment tool [42]	1 (4.3)

**Table 7:** Medication Optimisation outcome measure tools identified and their usage frequencies in the included studies (*n* = 23).

Domains	Tools	*N* (%)
Changes in concurrent pain medications	Patient chart review or medication records retrieved from electronic system [45, 46]	2 (8.6)
	Changes in pain medication survey [36]	1 (4.3)

**Table 8:** Cost effectiveness outcome measure tools identified and their usage frequencies in the included studies (*n* = 23).

Tools	*N* (%)
Incremental cost‐utility ratio (ICUR) and incremental cost‐effectiveness ratio (ICER) [32] – Decision tree and Markov model used for analysis. – ICUR was calculated per QALY gained, utilising EQ‐5D‐5L health questionnaires. – ICER was calculated per Numeric Rating Pain Score (NRS) reduction. – Resource consumption collected from a societal perspective.	1 (4.3)
Incremental cost‐effectiveness ratio (ICER) [41] – QALYs calculated from SF‐12 Health Survey assessments – Resource consumption assessed from a societal perspective.	1 (4.3)
Incremental cost‐effectiveness ratio (ICER) [47] – 12‐month Markov model used from payer and societal perspectives. – Measured QALYs from EQ‐5D‐5L questionnaire and Numerical Rating Scale (NRS) for pain severity. – Direct medical costs estimated based on a physician survey.	1 (4.3)

### 3.3 HRQoL

A total of 33 tools were identified to assess various domains of Health‐Related Quality of Life (HRQoL) across 23 studies, contributing to 64.7% of the total tools (51 tools) identified (Table 4).

#### 3.3.1 Physical Health

Studies assessed the impact on physical health by measuring pain intensity and its effect on sleep (Table 4). Five different tools were used to assess pain intensity in 13 studies (*n* = 13/23; 56.5%), with the Numeric Pain Rating Scale (NPRS) being the most widely used measure employed in studies (*n* = 7/23; 30.4%). The validation of this tool was confirmed in only two studies, and most of the studied conditions were related to musculoskeletal pain (*n* = 19/23; 82.6%). The Short‐Form McGill Pain Questionnaire (SF‐MPQ) was the second most used, employed in two studies (*n* = 2/23; 8.6%). Additionally, two specific pain assessment tools for neuropathic pain were used: the Neuropathic 4 Pain Questions (DN4) tool and the Pain Detect questionnaire. Both tools administered the questionnaires at baseline and at 3 months or beyond after providing the intervention.

The impact on sleep was assessed in four studies (*n* = 4/23; 17.3%) using three different tools: the Pain‐Related Sleep‐Interference Scale (PRSIS); the Medical Outcomes Study Sleep Scale (MOS‐Sleep); and the Chronic Pain Sleep Inventory.

#### 3.3.2 Psychological Health

In terms of assessing the cognitive and emotional response to pain, 7 tools were used in 13 studies (*n* = 13/23; 56.5%) (Table 4). The Hospital Anxiety and Depression Scale (HADS) was the most used tool, utilised in five studies (*n* = 5/23; 21.7%). Notably, opioid use was among the most used treatments in those studies.

The second most common tools were the Beck Depression Inventory (BDI) and the Pain Catastrophising Scale (PCS), both of which were utilised in two studies each (*n* = 2/23; 8.6%). Other tools assessing different psychological aspects like fear of movement, mental health, and pain acceptance are summarised in Table 4.

#### 3.3.3 Level of Independence

In terms of assessing performance status and disability, 8 different tools were utilised in 10 studies (*n* = 10/23; 43.4%). Notably, specific types of pain, such as neck pain, were assessed using the Neck Disability Index (NDI), while back pain was assessed using the Roland‐Morris Disability Questionnaire (RMDQ) and Oswestry Disability Index Survey.

#### 3.3.4 Social Relationships

In assessing the impact of the intervention on social life and responsibility, only one study utilised the Sheehan Disability Inventory (SDI) tool, which was used in refractory chronic neuropathic pain patients treated with pregabalin.

#### 3.3.5 Tools Assessing Multiple Domains of HRQoL

Nine multidimensional tools were commonly used to assess multiple domains across all included studies (*n* = 23/23; 100%) (Table 4). The Brief Pain Inventory (BPI) was the most frequently used tool (*n* = 9/23; 39.1%) to assess pain intensity and its impact on daily activities. Additionally, the European Quality of Life‐5 Dimensions (EQ‐5D) was employed in 5 studies (*n* = 5; 21.7%) to assess mobility, self‐care, usual activities, pain/discomfort, and anxiety/depression.

The American Pain Society Patient Outcome Questionnaire was included in both the patient experience domain and the multidimensional HRQoL tools (Table 4). The questionnaire assesses pain intensity, physical function, emotional well‐being, and patient experience, making it a valuable tool for capturing multiple aspects.

### 3.4 Patient Experience

Patient experience, including perspectives on healthcare, satisfaction, knowledge, and health status change, was assessed in 11 studies (*n* = 11/23; 47.8%) using 10 different tools (Table 5). It was noted that in some studies, researchers used multiple tools to assess patient experience. For instance, the Patient Satisfaction with Pain Management questionnaire was used together with the Barriers Questionnaire II [34], and similarly, the Patient Satisfaction Survey was used alongside the Pre‐ and Post‐Knowledge Assessment Questionnaire [45].

### 3.5 Medication‐Related Adverse Events

In total, 6 studies (*n* = 6/23; 26%) were dedicated to assessing adverse events (Table 6). Among them, four studies (*n* = 4/23; 17.3%) relied on patient‐reported adverse events or reviewed drug‐related adverse events in medical records. One study used a specific patient self‐administered questionnaire to assess side effects [49], while another study utilised the Screener and Opioid Assessment for Patients with Pain (SOAPP‐R) risk assessment tool to evaluate the risk of adverse events [42].

### 3.6 Medication Optimisation

Only three studies (*n* = 3/23; 13%) assessed changes in concurrent pain medications and medication optimisation using three tools (Table 7). The most used approach (*n* = 2/23; 8.6%) was patient chart review or medication records retrieved from electronic systems, where pharmacists were responsible for recording medication usage. Additionally, one study used a survey to assess changes in pain medication use [42].

### 3.7 Cost‐Effectiveness

Three studies were conducted to assess cost‐effectiveness (*n* = 3/23; 13%) (Table 8). In the first study, cost‐effectiveness was evaluated using the Incremental Cost‐Utility Ratio (ICUR) per Quality‐Adjusted Life Year (QALY) gained, based on EQ‐5D‐5L health questionnaires, and the Incremental Cost‐Effectiveness Ratio (ICER) per Numeric Rating Pain Score (NRS) reduction. These calculations were performed using a decision tree and Markov model, with resource consumption data collected from a societal perspective [32].

In the second study, the ICER was used to evaluate cost‐effectiveness, with Quality‐Adjusted Life Years (QALYs) calculated from Short Form‐12 Health Survey (SF‐12) assessments. Resource consumption was assessed from a societal perspective, considering direct medical and nonmedical costs in various categories [41].

In the third study, the ICER was employed to assess cost‐effectiveness through a cohort simulation based on a 12‐month Markov model from both payer and societal perspectives. The effectiveness measures included QALYs derived from the EQ‐5D‐5L questionnaire and the Numerical Rating Scale (NRS) for pain severity, with direct medical costs for pain severity levels estimated from a physician survey [47].

## 4 Discussion

The aim of this systematic review was to identify and evaluate outcome measures applied in primary care‐based pharmacotherapy pain management services provided by healthcare professionals, and the tools used to assess these outcomes. Although the findings are intended to be applied specifically in the Scottish context, the findings could aid in the evaluation of various primary care designs of pain management and support research and practice globally.

In this systematic review, 51 tools were identified to assess outcomes in chronic pain across the 23 included studies. Most studies examined only one to two outcomes (out of the five predefined outcomes). Health‐Related Quality of Life (HRQoL) was the most frequently assessed outcome, using the commonly employed tool, the Brief Pain Inventory (BPI), to evaluate both pain intensity and its impact on daily activities. On the other hand, medication optimisation and cost‐effectiveness were the least assessed outcomes. Furthermore, the tools used were inadequately described in 60.8% (*n* = 14/23) of the studies, and there was considerable variability in the timing and frequency of applying the measurement tools among studies (Supporting Information S4). Additionally, limited details were provided regarding the validation of the tools before their utilisation.

The results of this review demonstrated that assessing the HRQoL physical health outcome through pain intensity using the Numeric Pain Rating Scale (NPRS) tool was the most frequently evaluated domain and tool, which was consistent with several other reviews that investigated outcome measures in chronic pain [6, 7, 8, 53]. This finding aligned with the International Neuropathic Pain Special Interest Group (NeuPSIG) guidelines, which recommended the use of NPRS for evaluating pain intensity and treatment effectiveness [54].

In this review, it was found that most studies examined only one to two of the predefined outcomes. This aligns with findings from other systematic reviews assessing chronic musculoskeletal pain [7] and neuropathic pain [53]. This could possibly be driven by the complexity and multifaceted nature of chronic pain, which might make it challenging to comprehensively capture all relevant outcomes within a single study, and the need to minimise patient assessment burden, time constraints, and ease of administration [7]. This observation is supported by several studies investigating the utility and responsiveness of various pain measures, which reported that short and simple assessment tools were more responsive than more complex tools [55, 56]. However, a notable distinction in our review was that each of the included studies utilised at least one multidimensional tool (100%), covering multiple domains. In contrast, Litcher‐Kelly et al. and Dosenovic et al. employed multidimensional tools in less than 16% of the included studies, focusing on a single‐dimension target tool [7, 53]. This variation could be attributed to the type of included studies, which were controlled trials aiming primarily to assess changes in pain intensity and were confined to specific types of chronic pain (e.g., neuropathic, musculoskeletal pain), potentially limiting the range of applicable measures to those conditions. In contrast, this review included observational studies that capture a more real‐world evaluation, covering all types of chronic pain.

In this systematic review, 60.8% (*n* = 14) of the included studies lacked detail when describing the tools used (e.g., frequency of use, reasons for selection) and did not provide insights into the validation of the tools used (Table S5). This finding was consistent with other reviews assessing chronic pain outcome measures [7, 8]. By utilising validated measures, healthcare professionals can gain a better insight into the outcomes, enhancing the credibility and reliability of the study findings [35].

Cost‐effectiveness was one of the two least assessed outcomes in this review (13%), which was even lower (6%) in another systematic review conducted by Dosenovic et al. assessing outcome measures in neuropathic pain [53]. Limited inclusion of cost‐effectiveness can be attributed to factors such as the need for substantial financial resources and specialised expertise, prioritisation of clinical outcomes and patient experiences, complexity, time‐consuming nature of the analysis, and challenges with data availability on healthcare resource utilisation and costs [57].

### 4.1 Study Strengths and Limitations

In this systematic review, we acknowledge both strengths and limitations. The comprehensive search strategy accurately identified a wide range of chronic pain diseases using MeSH terms and keywords across different databases, along with hand searches to minimise the risk of missing relevant studies. We included all types of chronic pain and measures, assessed by healthcare providers or patients, as well as various timeframes for conducting interventions. Furthermore, this review underwent rigorous validation by two authors, including a quality assessment of all included studies, with a risk of bias assessment forming a key characteristic of a systematic review methodology. The quality of the identified studies was high (13 of the 15 cohort studies and 7 of the 8 cross‐sectional studies scored as high quality), showing that the outcomes extracted came from high‐quality studies.

The consensus outcome framework of pharmacotherapy services in Scotland was adopted as the basis for assessing outcomes in this review [9], however there are other reviews [6, 53] assessing chronic pain which use the Initiative on Methods, Measurement, and Pain Assessment in Clinical Trials (IMMPACT) framework [58]. The IMMPACT framework was not considered in this study due to its primary focus on clinical trials, whereas this review concentrated on observational studies. Furthermore, the Scottish consensus framework covered all the outcome areas within the IMMPACT framework with addition of medication optimisation and cost‐effectiveness domains. Moreover, the five‐predefined outcomes adopted in this systematic review was developed by pharmacists experienced in delivering pharmacotherapy services in varied clinical conditions beyond but including chronic pain management providing the potential for applicability of these findings to other clinical service areas. We acknowledge that the inclusion of clinical trials could have provided further insights, especially regarding cost‐effectiveness outcomes, but it may have limited the overall assessment scope to treatment‐related targets (e.g., pain intensity). However, this review aimed to identify tools being used in routine clinical practice hence the focus on assessment of observational studies with the goal to inform potential tools to adopt as part of a pharmacotherapy service in primary care. Moreover, we recognise that although cohort studies provide an insight into clinical practice, their nature as research studies may not necessarily provide an accurate reflection of everyday clinical practice. Finally, limited descriptions of assessment tools in some articles posed challenges in identifying the main domain target, but these challenges were addressed by conducting additional literature searches for clarification.

### 4.2 Future Research and Implications

This research can be beneficial for researchers and healthcare providers by providing a comprehensive list of available tools used in observational studies classified according to domain coverage. It aids in choosing the most suitable tool for specific study settings. To further advance this field, validation of the identified tools should be conducted rigorously to ensure greater consistency and standardised assessments of chronic pain services. The findings of this work intend to be integrated within the Scottish primary care system to evaluate the outcomes of pharmacy‐led pain clinics, which is of importance considering the novelty of the delivery model. However, the findings can be applied throughout any primary care setting globally to inform evidence‐based evaluations of pain management and support and may provide useful cross‐comparison between different delivery models. Furthermore, future research could incorporate nonpharmacological alongside pharmacological to provide a more comprehensive perspective on chronic pain management in outpatient settings.

The International Consortium for Health Outcomes Measurement (ICHOM) focuses on defining global patient‐centred outcome measures in various disease conditions, and creating standard outcome sets for clinical conditions and certain populations [59]. However, general chronic pain measures have not yet been included in their scope, and this study could provide a starting point for identifying the best outcome measures for use in chronic pain.

## 5 Conclusions

This review highlights gaps in enabling a more holistic assessment of pharmacotherapy services, given the diversity of outcome measures being utilised. It underscores the need for enhanced consistency by validating the identified tools and adopting standardised tools in clinical practice. Implementing such tools can significantly improve the quality of pharmacotherapy service assessments and, ultimately, contribute to more effective patient care and improved healthcare outcomes.

## Author Contributions

Marion Bennie, Rosemary Newham and Natalie Weir were responsible for the overall research programme conceptualisation for this work. Marion Bennie, Emma Dunlop and Natalie Weir developed the study aim. Abdulrahman Sharaf with Marion Bennie, Emma Dunlop and Natalie Weir developed the study methodology and search strategy. Abdulrahman Sharaf conducted the literature search and analysed the data. Sumaya Alsalah, Natalie Weir and Emma Dunlop performed validation of the work. Abdulrahman Sharaf wrote the draft manuscript, with contribution from Emma Dunlop, Natalie Weir, Rosemary Newham and Marion Bennie.

## Conflicts of Interest

The authors declare no conflicts of interest.

## Supporting information

Supporting information.

Supporting information.

Supporting information.

Supporting information.

Supporting information.

## Data Availability

The authors have nothing to report.
