# Supplementary material for: Systematic Review of Outcome Measures in Pharmacologically Managed Chronic Pain: Informing a New Outcome Framework for Healthcare Provider‐Led Pharmacotherapy Services
Source: J Eval Clin Pract. 2025 Feb 26;31(2):e70029. doi: 10.1111/jep.70029 (PMC11865632; doi:10.1111/jep.70029)
Supplement: Supplementary file 1 — Supporting information. [file JEP-31-0-s001.docx]

**Table S1- PICO model**

|  | **Inclusion Criteria** | **Exclusion Criteria** |
| --- | --- | --- |
| **Population** | Patients with non-malignant chronic pain (pain lasting more than 3 months), including: primary chronic pain not attributed to other conditions; or secondary chronic pain resulting from an underlying disease or condition. | Patients with chronic cancer pain or acute pain. |
|  | Age: >18 years old . | Patients < 18 years old. |
|  | Setting: Outpatient services (non-hospitalised patients). | Hospitalised patients |
|  | Study types including observational studies (cohort studies, case-control studies, and cross-sectional studies). | Clinical trials, case reports, case series, and narrative reviews, expert opinion, abstracts, conference papers, and study protocols. |
| **Intervention** | Professional-led pain management program using: acetaminophen; analgesics; NSAIDS; opioids; antidepressants; anti-epileptics; and topical capsaicin/rubefacients/lidocaine. | Interventions focused only on non-pharmacological approaches or had a preventive purpose (e.g., pre-operative pain control). |
|  | Healthcare professionals involved in the assessment and management of chronic pain such as: physicians; pharmacists; nurses; physiotherapists. | Professionals not directly related to chronic pain management. |
|  | Outpatient services including: community settings; primary care; rehabilitation centres; pain clinics; general practitioners' offices; outpatient clinics; care home settings | Inpatient setting e.g.: Secondary or tertiary care hospital |
| **Comparison** | Existing outcome measures utilised in chronic pain management | No outcome measures utilised in chronic pain management |
| **Outcomes** | Measures for outcomes associated with chronic pain including: patient experience; medication-related adverse events; cost-effectiveness; medication optimisation; health-related quality of life. Outcome measures should be assessed by healthcare providers or through patient self-report | Measures assessing other than the predefined outcomes |
