# Supplementary material for: Systematic Review of Outcome Measures in Pharmacologically Managed Chronic Pain: Informing a New Outcome Framework for Healthcare Provider‐Led Pharmacotherapy Services
Source: J Eval Clin Pract. 2025 Feb 26;31(2):e70029. doi: 10.1111/jep.70029 (PMC11865632; doi:10.1111/jep.70029)
Supplement: Supplementary file 2 — Supporting information. [file JEP-31-0-s003.docx]

| **No** | **Searches** | **Results** |
| --- | --- | --- |
| **MEDLINE** | | |
| 1 | chronic pain.mp. or exp Chronic Pain/ | 58240 |
| 2 | Low Back Pain/ or Pain/ or Back Pain/ or Musculoskeletal Pain/ or Neck Pain/ or Nociceptive Pain/ | 207344 |
| 3 | 1 or 2 | 248427 |
| 4 | paracetamol.mp. or exp Acetaminophen/ | 26656 |
| 5 | exp analgesics/ or analgesics, non-narcotic/ or analgesics, short-acting/ or narcotics/ | 587628 |
| 6 | nsaid*.mp. or exp Anti-Inflammatory Agents, Non-Steroidal/ | 224998 |
| 7 | opioid*.mp. or exp Analgesics, Opioid/ | 202744 |
| 8 | exp Antidepressive Agents/ or antidepressant*.mp. | 189774 |
| 9 | exp Anticonvulsants/ or antiepileptic*.mp. | 163262 |
| 10 | exp Antidepressive Agents/ or antidepressant*.mp. | 189774 |
| 11 | 4 or 5 or 6 or 7 or 8 or 9 or 10 | 946305 |
| 12 | pain assessment.mp. or exp Pain Measurement/ | 97723 |
| 13 | pain management.mp. or exp Pain Management/ | 64856 |
| 14 | 12 or 13 | 149120 |
| 15 | outpatient.mp. or exp Outpatients/ | 183014 |
| 16 | exp Primary Health Care/ or ''primary care''.mp. | 276059 |
| 17 | pain clinic.mp. or exp Pain Clinics/ | 3462 |
| 18 | exp Community Health Services/ or community service*.mp. | 334360 |
| 19 | 15 or 16 or 17 or 18 | 747885 |
| 20 | 3 and 11 and 14 and 19 | 1572 |
| 21 | 20 Limit by abstract in the last 10 years | **900** |
| **EMBASE** | | |
| 1 | ''chronic pain''.mp. or exp chronic pain/ | 102170 |
| 2 | foot pain/ or neck pain/ or ankle pain/ or jaw pain/ or hip pain/ or abdominal pain/ or neuropathic pain/ or low back pain/ or eye pain/ or eyelid pain/ or "headache and facial pain"/ or musculoskeletal pain/ or chronic inflammatory pain/ or epigastric pain/ or bone pain/ or musculoskeletal chest pain/ or lower abdominal pain/ or nociceptive pain/ or visceral pain/ | 425835 |
| 3 | 1 or 2 | 501139 |
| 4 | exp pain assessment/ or exp pain measurement/ or exp pain parameters/ or exp pain severity score/ | 275186 |
| 5 | ''pain management''.mp. | 51438 |
| 6 | 4 or 5 | 310110 |
| 7 | paracetamol.mp. or exp paracetamol/ or acetaminophen.mp. | 116621 |
| 8 | analgesic*.mp. or exp analgesic agent/ or narcotic analgesic agent/ or exp analgesic activity/ or exp antipyretic analgesic agent/ | 1104355 |
| 9 | exp celecoxib/ or exp naproxen/ or exp diclofenac/ or exp acetylsalicylic acid/ or exp indometacin/ or exp cyclooxygenase 2 inhibitor/ or exp nonsteroid antiinflammatory agent/ or nsaid*.mp. or exp ibuprofen/ | 1154874 |
| 10 | (narcotic* or analgesic*).mp. [mp=title, abstract, heading word, drug trade name, original title, device manufacturer, drug manufacturer, device trade name, keyword heading word, floating subheading word, candidate term word] | 238445 |
| 11 | antidepressant*.mp. or exp antidepressant agent/ or exp tricyclic antidepressant agent/ or exp tetracyclic antidepressant agent/ | 560948 |
| 12 | phenobarbital/ or topiramate/ or exp anticonvulsive agent/ or valproic acid/ or antiepileptic*.mp. or carbamazepine/ or phenytoin/ | 465059 |
| 13 | 7 or 8 or 9 or 10 or 11 or 12 | 2394879 |
| 14 | exp outpatient care/ or outpatient.mp. or exp outpatient/ | 382581 |
| 15 | ''primary care''.mp. or exp primary medical care/ | 229879 |
| 16 | exp community program/ or exp community care/ or exp community/ | 210124 |
| 17 | 14 or 15 or 16 | 786604 |
| 18 | 3 and 6 and 13 and 17 | 2321 |
| 19 | 18 Limit by abstract and last 10 years | **1594** |
| **CINHAL** | | |
| S1 | AB (MH "Chronic Pain") OR "chronic pain" OR (MH "Nociceptive Pain") OR (MH "Visceral Pain") OR (MH "Shoulder Pain") OR (MH "Muscle Pain") OR (MH "Knee Pain") OR (MH "Eye Pain") OR (MH "Chest Pain") OR (MH "Back Pain") OR (MH "Low Back Pain") OR (MH "Elbow Pain") OR (MH "Abdominal Pain") OR (MH "Musculoskeletal Pain") | 91,245 |
| S2 | AB (MH "Pain Management") OR "pain management" OR (MH "Pain Management Nursing") OR (MH "Pain Management Nurses") OR (MH "Pain Measurement") OR "pain assessment" | 73,890 |
| S3 | AB (MH "Analgesics, Opioid+") OR (MH "Narcotics+") OR (MH "Analgesics+") OR "analgesic*" OR (MH "Acetaminophen") OR (MH "Antiinflammatory Agents, Non-Steroidal+") | 119,777 |
| S4 | AB "anticonvulsant" OR (MH "Anticonvulsants+") OR (MH "Antidepressive Agents+") OR ("Antidepressant*") | 51,975 |
| S5 | S3 OR S4 | 165,286 |
| S6 | AB (MH "Outpatient Service") OR (MH "Outpatients") OR "outpatient" OR (MH "Primary Care Nurse Practitioners") OR (MH "Primary Health Care") OR "Primary care" OR (MH "Community Health Services") OR "community services" OR (MH "Community Health Nursing") OR (MH "Pain Clinics") OR "pain clinic" | 249,942 |
| S7 | S1 AND S2 AND S5 AND S6 | 505 |
| S8 | S7 limit Last 10 years | **339** |
| **TOTAL** | | **2833** |
