# Supplementary material for: Systematic Review of Outcome Measures in Pharmacologically Managed Chronic Pain: Informing a New Outcome Framework for Healthcare Provider‐Led Pharmacotherapy Services
Source: J Eval Clin Pract. 2025 Feb 26;31(2):e70029. doi: 10.1111/jep.70029 (PMC11865632; doi:10.1111/jep.70029)
Supplement: Supplementary file 3 — Supporting information. [file JEP-31-0-s002.docx]

**Table S3:** Baseline studies characteristics

| Study ID | Summary of Included Studies | | | | | | | | |
| --- | --- | --- | --- | --- | --- | --- | --- | --- | --- |
|  | **Country** | **Study design** | **Sample size(n)** | **Aim** | **Population** | **Intervention** | **Comparison** | **Outcomes** | **Key findings** |
| (Nadkarni et al., 2022) | India | prospective, observational Cohort study | 100 | To determine the prescribing pattern for neuropathic pain and to assess the improvement and tolerability of the prescribed medications in neuropathic pain | either gender aged 18-65 years with newly diagnosed neuropathic pain | First line agents for neuropathic pain either as monotherapy or in combination | combination therapy VS monotherapy | assess the pain improvement and tolerability | 1. response to combination therapy with 1st line drugs were better when compared to monotherapy in managing of pain. 2. The response to NP was higher in subjects with younger age group, good socio-economic status, familial support, good awareness and functional status, regular follow-up visits. 3. Poor response was related to advancing age, lack of family/social support, multiple illnesses and medications and adverse effects. |
| (Robinson et al., 2022) | USA | Cross-Sectional Survey | 572 patients and 153 physicians participants | aimed to determine the factors associated with satisfaction in US patients and their physicians. | physicians and their patients diagnosed with and consulting for OA in the USA | medication for osteoarthritis | Patient VS physician Reported Overall Satisfaction With Medication For Osteoarthritis | •Patient Reported Overall Satisfaction With Medication For Osteoarthritis •Physician Rated Overall Satisfaction With Medication For Osteoarthritis •Patient Reported Expectation Of Effectiveness Of Medication For Osteoarthritis | Although efficacy was strongly associated with both patients’ and physicians’ satisfaction with medication, other factors were also important, including exercise (for patients), tolerability (for physicians), and knee OA (for physicians). |
| (Zinboonyahgoon et al., 2023) | Thailand | Prospective observational Cohort study | 29 | To evaluate the cost-effectiveness and cost-utility of additional SCS to conventional management (CMM) in patients with chronic refractory pain in Thailand. | patients with chronic refractory pain in Thailand | spinal cord stimulation (SCS) and conventional management (CMM) | (SCS + CMM) VS (CMM) | •Pain Reduction •Improvement Of Function •Quality Of Life •Cost Effectiveness •Cost Utility •Qaly Gained •Numeric Rating Pain Score (Nrs) Reduction | Spinal cord stimulation is effective in pain control and improves the quality of life for patients with chronic refractory pain. However, the ICUR of SCS is above the WTP, leading to the interpretation that SCS is still not a cost-effective treatment in the current context in Thailand. |
| (Moreira de Barros et al., 2021) | Brazil | noninferiority, retrospective observational cohort single center study | 262 | aimed to provide a real-world evidence for the analgesic use of methadone, compared with morphine | age >18 years, diagnosed with chronic pain (lasting longer than 6 months), undertreatment with methadone or morphine, no concomitant treatment with other opioids | Methadone, Morphine | Methadone VS Morphine | 1. Analgesic Effect. 2.Side Effects. 3. pain intensity | Methadone was superior to morphine in a 20% noninferiority margin for reducing worst pain. |
| (Ganguly et al., 2021) | USA | cross-sectional Patient-reported survey study | 166 | To examined racial/ethnic differences in patient satisfaction and barriers to pain management among a cohort of PWH receiving LTOT | (1) ≥18 years of age, (2) diagnosis of HIV infection, (3) receipt of ≥3 opioid prescriptions ≥21 days apart within a 6 month period in the prior year, and (4) attendance at least 1 visit to the study clinical sites within the prior 18 months | long term opioid therapy at a chronic opioids dose | Non-white and White HIV patients | •Satisfaction With Pain Management (0-10) •Patient Related Barriers To Pain Management, Including Patient Perceptions Of Pain Medications, Fatalism, And Communication About Pain | Non-white participants were noted to receive chronic opioids for a shorter mean duration of time than white participants and lower mean morphine equivalent daily dose (MEDD) than white participants. In adjusted analyses, there was no significant difference in satisfaction with pain management among non-white and white participants. There was no significant difference in barriers to pain management in unadjusted nor adjusted models. |
| (Gudin et al., 2020) | USA | Prospective observaional study (Survey) | 199 | "Evaluating topical analgesic pain-relieving patch in reducing pain severity and improving function" | "ages 18 to 64 years, received a topical pain-relieving patch from their treating physician and had been diagnosed with a mild to moderate pain condition" | "OTC topical pain-relieving patch, the Salonpas® Pain Relieving Patch ( containing methyl salicylate 10%, menthol 6%, and camphor 3.1% )" | control group | "*Pain intensity and interference *patient satisfaction * Changes in Concurrent Pain Medications *Side effect " | "*In the treatment group (TG), pain severity and interference scores decreased by 49% and 58.1%, respectively, after 14 days of using the pain-relieving patch. *60.5% of the TG reported reduced use of oral pain medications, and 90.8% were highly satisfied with the patch. *No side effects of the treatment were reported, indicating the safety of the pain-relieving patch." |
| (Kaboré et al., 2020) | Canada | retrospective cohort study | 160 | "to identify characteristics of individuals who are most likely to benefit from long-term opioid therapy in terms of reduction in pain severity and improved mental health–related quality of life (mQoL) without considering potential risks." | "they did not report opioid use in the past 6 months before the initial visit to the pain clinic" | opioids | improvers VS non-improvers | pain severity and mQoL. | "Clinically significant reduction in pain severity was observed in 26.7% of patients, while improvement in mQoL was reported by 20.2% of patients on long-term opioid therapy. and alcohol or drug problems were weakly associated with pain severity at 12-month follow-up." |
| (Ramírez-Maestre et al., 2020) | Spain | cross-sectional | 675 | (1) To identify the individual factors that differentiate patients who had been prescribed opioids for the management of chronic back pain from those who had not been prescribed opioids and (2) to determine which factors make significant and independent contributions to the prediction of opioid prescribing. | Patients experiencing back pain and had been experiencing pain for at least the last 3 months | opioid analgesics, given as an opioid pain medication or nonopioid analgesics. | "Participants Prescribed Opioids VS Participants not Prescribed Opioids" | pain intensity, depressive symptoms, pain catastrophizing, and pain acceptance | Although no differences were found between men and women, participants with chronic noncancer pain who were prescribed opioids were older, reported higher levels of pain intensity and depressive symptoms, and reported lower levels of pain-acceptance. An independent association was found between pain intensity and depressive symptoms and opioid prescribing. |
| (Sicras-Mainar et al., 2020) | Spain | non-interventional, retrospective, Cohort longitudinal study | 38,539 | to analyze health outcomes, resource utilization, and costs in osteoarthritis patients with chronic nociceptive pain who began treatment with an opioid in real-world practice | (a) patients aged ⩾18 years starting treatment with a new opioid for the treatment of osteoarthritis for the first time with chronic nociceptive pain of more than 3 months | opioid drugs | Knee/hip VS spine. VD other OA patients | •Health/Non Health Resource Utilization •Costs •Treatment Adherence •Pain Change •Cognitive Functioning •Dependence For Basic Activities Of Daily Living (Badl) | " A total of 19.1% of patients remained on initial opioid at 36 months, without significant differences by osteoarthritis site . Mean total adjusted cost was €17,915, with 27.7% corresponding to healthcare resources and 72.3% to lost productivity. Hospital admissions for osteoarthritis-related surgical interventions accounted for 15.8% of total healthcare cost. A slight mean pain reduction was observed: –1.3 points, –16.9%, with increases in cognitive deficit and moderate to total dependence for BADL in a median duration of opioid use of 203 days." |
| (Lee et al., 2020) | USA | Cross-sectional survey | 178 | To examines the differential effects of pain relief on patient satisfaction based on patients’ perceptions about pain management. | adults aged 18 years and older who were US residents and had Internet access at the time of assessment. | patients’ confidence in their physician’s pain management | N/A | •Patient Satisfaction With Pain Care •Patient Satisfaction With Pain Relief •Perceptions About Participation In Treatment Decisions •Confidence In Physicians | patients reported moderate patient satisfaction with their pain care . Among patients who reported low pain relief, the level of pain relief and con- fidence in their physicians were positively associated with satisfaction after adjusting for other covariates and control variables. Among patients who reported high pain relief, the level of pain relief positively predicted satisfaction after adjusting for other covariates and control variables. Patients’ confidence in their physicians positively moderated the effects of pain relief on satisfaction. |
| (Taguchi et al., 2019) | Japan | non-interventional, multicenter, observational prospective cohort study | 360 | to evaluate the effectiveness of pregabalin for treatment of patients with chronic cervical pain and accompanying upper limb radiating pain (NeP component) | patients aged ≥20 years with chronic cervical pain and accompanying radiating pain to superior limb(s) with a NeP component of ≥12 weeks in duration at baseline | pregabalin therapy | Usual Care | "•Pain Related Sleep Interference Scale (Prsis) •Pain Numerical Rating Scale (Nrs) •Neck Disability Index (Ndi) " | "1. Least-squares mean change in PRSIS from baseline to week 8 favored pregabalin. 2. Similar observations were seen at week 4 in favor of pregabalin versus usual care. 3. Pregabalin significantly improved pain NRS and NDI scores at weeks 4 and 8.and improved QoL versus usual care. 5. Pregabalin was generally well tolerated." |
| (Wayne et al., 2019) | Boston, USA | Observational prospective cohort study | 309 | To report the results of health economic analyses comparing two treatment approaches for chronic low back pain (CLBP). | Participants were ‡21 years of age and En- glish speaking had nonspecific CLBP or had herniated nucleus pulposus or stenosis | Integrative or conventional care for CLBP as prescribed by the treating clinician(s) | Osher Clinical Center VS Non-Osher Clinical Center | Quality-adjusted life years (QALYs). Cost per QALY using an incremental cost-effectiveness ratio (ICER). ICERs based on CLBP-specific effectiveness measures (Roland Disability Questionnaire [RDQ] and bothersomeness of pain [BOP]) were exploratory outcomes. | When adjusted for baseline differences, self-reported costs were higher in the OCC group with only small effects on QALYs. However, trends toward decreased direct expenditures and medication usage over time warrant further investigation. Future studies evaluating potential benefits of integrative care models for the management of CLBP should employ randomized designs, longer observational periods, and explore multiple metrics of cost-effectiveness. |
| (McCann et al., 2018) | USA | retrospective cohort study | 29 | The primary goal was to determine whether the program affected the number of NMCP patients who continued to use opioid medications. The secondary goal was to compare those who continued opioid medications to those who chose to wean off them | patients with NMCP on opioid medications | are system of opioid medication management | remain on opioids VS wean off | Whether Patients Elected To Remain On Opioids, Wean Opioids, Or Transfer Care •Evaluation Of Pain •Evaluation Of Functional Status •Evaluation Of Mood | 1. 38% elected to wean opioids, 53% continued opioid medication, and 9% transferred care. Mean morphine equivalent mg/day was the prime determinant for ability to wean (17.01 mg/day) compared with maintaining (30.61 mg/day) (P = .0397; CI, 0.68 to 26.51). Patients maintaining opioid treatment showed no statistically significant change in any measured data point from beginning until end of the evaluation period. |
| (Elsesser & Cegla, 2017) | Germany | cross sectional observational study | 333 | To investigate the outcomes of long-term opioid therapy compared to nono- pioid treatment in CNCP patients | age above 18 years, fluency in Ger man, and pain duration of at least 6 months as NCCP | The long term opioid therapy compared to nonopioid treatment given as opioids (doses not specified) and nonopioid analgesics for at least 3 months. | patients with continuous opioid treatment VS patients receiving nonopioid analgesics | measures of pain, functional disability, psychological wellbeing, and quality of life (QoL). | 1. The opioid and nonopioid groups did not differ with regard to pain intensity or satisfaction with analgesic. 2. Patients with continuous opioids treatment reported higher neuropathic like pain, longer duration of pain disorder, lower functional level, wellbeing, and physical QoL in comparison to patients receiving nonopioid analgesics. 3. patients on high potency opioids reported more psychological impairment than patients on low potency opioids but no advantage with regard to pain relief. |
| (Ghodke et al., 2018) | USA | retrospective cohort observational study | 171 | "To examine the effectiveness of short- acting opioids (SAO) vs long-acting opioids (LAO) and combination therapies (SAO and LAO) for treat- ing chronic osteoarthritis pain in a retrospective trial." | diagnosis of moderate to severe osteoarthritis, and receipt of opioid prescriptions for chronic pain management for more than six months | short acting opioids (SAO) vs long acting opioids (LAO) and combination therapies (SAO and LAO) for treating chronic osteoarthritis pain | short acting opioids (SAO) vs long acting opioids (LAO) and combination therapies (SAO and LAO) for treating chronic osteoarthritis pain | "•Average Pain Scores •Lowest Pain Scores •Morphine Equivalence " | "There was no statistical difference be- tween reported average and low pain scores for the SAO vs LAO groups, although the SAO group on average had a significantly lower morphine equivalence. No significant differences in pain scores existed when comparing covariates for the SAO vs LAO groups. " |
| (Vogler et al., 2017) | USA | prospective cohort design | 35 | evaluates the impact of pain education group visits on patients with chronic non-cancer pain (CNCP) | patients had a diagnosis of CNCP, age of at least 18 years old | pain education group visits | control group | •Functional Status (Measured By The Pain Intensity, Enjoyment Of Life, And General Activity (Peg) Score And The Oswestry Disability Index) •Knowledge •Behavior (Opioid Use And Utilization Of Emergency And Urgent Care Services) •Satisfaction | A moderately positive correlation was observed between PEG and Oswestry Disability Index (r = 0.47, p < 0.05). Patient knowledge improved after watching the videos and participating in group discussion. The median amount of daily morphine equivalents for patients (n = 14) was 17.5mg (range 0-120mg) at the first study, and decreased to 12.5mg (range 0-110mg) at the last study visit (p = 0.39). None of the 14 patients utilized emergency or urgent care services for pain during the study. Overall, patient satisfac- tion scores with the intervention were high. |
| (White et al., 2018) | Canada | single-group, pretest–posttest analysis (Questionnaire ) | 102 | The purpose of this study was to observe the clinical outcomes of people who completed a 6-week outpatient interdisciplinary pain management programme and to determine whether a change in pain knowledge predicts these clinical outcomes | people in Vancouver who completed a 6-week outpatient interdisciplinary pain management programme. | interdisciplinary pain management programme | pre and post application of the program | (pain interference, pain severity, depression, and opioid intake) | Participants experienced significant improvements in pain knowledge, pain interference, pain severity, depression, and opioid intake between intake assessment and discharge, but change in pain knowledge did not significantly predict any clinical outcomes. |
| (Igarashi et al., 2015) | Japan | cohort study | pregabalin, n=157; usual care, n=174 | To assess the cost-effectiveness of pregabalin for the treatment of chronic low back pain with accompanying neuropathic pain (CLBP-NeP) from the health care payer and societal perspectives. | "• Mean age: 60 years. • Patients with refractory pain after 3 months of (NSAID) treatment. • Patients with no bladder or rectal disturbance. • Patients with no contraindication for any medication. • Patients with no leg paralysis in the study period." | pregabalin | pregabalin VS usual care | •Quality Adjusted Life Years (Qalys) •Direct Medical Costs •Hospitalization Costs •Productivity Losses | Direct medical costs and hospitalization costs were both lower in the pregabalin arm compared with usual care. |
| (Dunn et al., 2014) | USA | A one-time self-report survey | 227 | " Evaluate the presence of pain and treatment for pain within a large sample of patients being maintained on methadone for the treatment of opioid use disorder" | methadone-maintaned patients | Methadone | N/A | Pain intensity | "*Mean BPI severity score was 5.7 and BPI interference score was 5.4 out of 10, indicating moderate pain and its impact on daily functioning, respectively. *Age and methadone dose were found to be significantly associated with having pain. Pain was a significant predictor of benzodiazepine use. *Only 13% (N = 18) of patients with pain were receiving pain management, and few were being treated with nonopioid adjuvant analgesics. *Patients who received treatment reported a mean 51% improvement in their pain, suggesting they are not treatment refractory." |
| (Jouini et al., 2014) | Canada | cohort study | 486 | Describe the pharmacotherapeutic management of primary-care patients with chronic noncancer pain, assess their satisfaction with pain treatment, and identify the determinants of their satisfaction. | 1) aged at least 18 years; 2) suffering from CNCP, defined as pain lasting for 6 months or more and not related to cancer; 3) reporting an average pain intensity in the past 7 days of at least 4 on a 0–10 intensity scale where 0 means “no pain” and 10 means “worst possible pain”; 4) suffering from pain at least 2 days a week. | analgesic effectiveness | before and after the use of analgesics | "•Pain Intensity •Emotional Well Being •Satisfaction With Pain Treatment •Barriers/Beliefs/Attitudes About Pain And Its Treatment " | The overall treatment satisfaction score decreased with more pain disability, probable depression and anxiety, more barriers to pain treatment, higher incidence of nausea, and use of over-the-counter analgesics. |
| (Ashworth et al., 2013) | UK | prospective cohort study | 715 | to explore the relationship between prescribed opioids and disability among patients consulting in primary care with back pain | patients have chronic pain | opioid prescription | patients with no opioids VS patients taking low/ medium/ high | The primary outcome was disability at 6 months | findings indicate that even after adjusting for a substantial number of potential con- founders, opioids were associated with slightly worse functioning in back pain patients at 6-month fol- low-up. |
| (Pérez et al., 2013) | Spain | multicenter, observational, prospective cohort study | 1845 | To describe clinical and resource utilization patterns in patients with refrac- tory neuropathic pain (NeP) who were prescribed pregabalin for the first time | adult patients with refractory chronic neuropathic pain of at least 6-months duration with poorer psychiatric conditions | pregabalin and analgesics | "pregabalin as monotherapy VS added pregabalin to their exist- ing pain treatments VS other pain treat- ments were prescribed" | •Self Reported Pain Intensity •Disability •Sleep Disturbances •Symptoms Of Anxiety And Depression •Health Related Quality Of Life (Hrqol) •Health Care Resource Utilization •Corresponding Costs | "1. Baseline differences: Pain intensity, patient disability, severity of depressive symptoms, and HRQoL showed significant differences. 2. Among the three treatment groups, no significant differences were reported for anxiety severity or sleep disturbances. 3. Subjects receiving add-on pregabalin utilized more direct and indirect resources, resulting in higher quarterly overall costs per patient." |
| (Blanco Tarrio et al., 2013) | Spain | observational multicenter prospective cohort study | 1,670 | To assess the effectiveness of pregabalin therapy in patients with NP refractory to previous treatments. | patients who had received no treatment or had been treated with a drug other than pregabalin during the previous 3 months | pregabalin therapy | Different pain etiology duration | •Pain Intensity •Interference Of Pain With Activities •Number Of Days With No Or Mild Pain •Treatment Satisfaction | At 3 months, pain intensity and its interference with activities decreased by half, while the number of days with no or mild pain increased by a mean of 4.5 days. Treatment satisfaction increased two fold. |

Ashworth, J., Green, D. J., Dunn, K. M., & Jordan, K. P. (2013). Opioid use among low back pain patients in primary care: Is opioid prescription associated with disability at 6-month follow-up? *Pain*, *154*(7), 1038-1044. <https://doi.org/10.1016/j.pain.2013.03.011>

Blanco Tarrio, E., Gálvez Mateos, R., Zamorano Bayarri, E., López Gómez, V., & Pérez Páramo, M. (2013). Effectiveness of pregabalin as monotherapy or combination therapy for neuropathic pain in patients unresponsive to previous treatments in a Spanish primary care setting. *Clin Drug Investig*, *33*(9), 633-645. <https://doi.org/10.1007/s40261-013-0116-7>

Dunn, K. E., Brooner, R. K., & Clark, M. R. (2014). Severity and interference of chronic pain in methadone-maintained outpatients. *Pain Med*, *15*(9), 1540-1548. <https://doi.org/10.1111/pme.12430>

Elsesser, K., & Cegla, T. (2017). Long-term treatment in chronic noncancer pain: Results of an observational study comparing opioid and nonopioid therapy. *Scand J Pain*, *17*, 87-98. <https://doi.org/10.1016/j.sjpain.2017.07.005>

Ganguly, A. P., Lira, M. C., Lodi, S., Forman, L. S., Colasanti, J. A., Williams, E. C., Liebschutz, J. M., Del Rio, C., Samet, J. H., & Tsui, J. I. (2021). Race and satisfaction with pain management among patients with HIV receiving long-term opioid therapy. *Drug Alcohol Depend*, *222*, 108662. <https://doi.org/10.1016/j.drugalcdep.2021.108662>

Ghodke, A., Barquero, S., Chelminski, P. R., & Ives, T. J. (2018). Short-Acting Opioids Are Associated with Comparable Analgesia to Long-Acting Opioids in Patients with Chronic Osteoarthritis with a Reduced Opioid Equivalence Dosing. *Pain Med*, *19*(11), 2191-2195. <https://doi.org/10.1093/pm/pnx245>

Gudin, J. A., Dietze, D. T., & Hurwitz, P. L. (2020). Improvement of Pain and Function After Use of a Topical Pain Relieving Patch: Results of the RELIEF Study. *J Pain Res*, *13*, 1557-1568. <https://doi.org/10.2147/jpr.S258883>

Igarashi, A., Akazawa, M., Murata, T., Taguchi, T., Sadosky, A., Ebata, N., Willke, R., Fujii, K., Doherty, J., & Kobayashi, M. (2015). Cost-effectiveness analysis of pregabalin for treatment of chronic low back pain in patients with accompanying lower limb pain (neuropathic component) in Japan. *Clinicoecon Outcomes Res*, *7*, 505-520. <https://doi.org/10.2147/ceor.S89833>

Jouini, G., Choinière, M., Martin, E., Perreault, S., Berbiche, D., Lussier, D., Hudon, E., & Lalonde, L. (2014). Pharmacotherapeutic management of chronic noncancer pain in primary care: lessons for pharmacists. *J Pain Res*, *7*, 163-173. <https://doi.org/10.2147/jpr.S56884>

Kaboré, J. L., Saïdi, H., Dassieu, L., Choinière, M., & Pagé, M. G. (2020). Predictors of Long-Term Opioid Effectiveness in Patients With Chronic Non-Cancer Pain Attending Multidisciplinary Pain Treatment Clinics: A Quebec Pain Registry Study. *Pain Pract*, *20*(6), 588-599. <https://doi.org/10.1111/papr.12883>

Lee, S., Smith, M. L., Dahlke, D. V., Pardo, N., & Ory, M. G. (2020). A Cross-Sectional Examination of Patients' Perspectives About Their Pain, Pain Management, and Satisfaction with Pain Treatment. *Pain Med*, *21*(2), e164-e171. <https://doi.org/10.1093/pm/pnz244>

McCann, K. S., Barker, S., Cousins, R., Franks, A., McDaniel, C., Petrany, S., & Riley, E. (2018). Structured Management of Chronic Nonmalignant Pain with Opioids in a Rural Primary Care Office. *J Am Board Fam Med*, *31*(1), 57-63. <https://doi.org/10.3122/jabfm.2018.01.170163>

Moreira de Barros, G. A., Baradelli, R., Rodrigues, D. G., Toffoletto, O., Domingues, F. S., Gayoso, M. V., Lopes, A., Barros Afiune, J., & Nunes Guimarães, G. M. (2021). Use of methadone as an alternative to morphine for chronic pain management: a noninferiority retrospective observational study. *Pain Rep*, *6*(4), e979. <https://doi.org/10.1097/pr9.0000000000000979>

Nadkarni, S., Ramesh, J., Bk, A., & Girish, K. (2022). A Prospective Observational Study On Pattern Of Drug Use For Neuropathic Pain In A Tertiary Hospital. *Research Journal of Pharmaceutical, Biological and Chemical Sciences*, *13*, 101-109. <https://doi.org/10.33887/rjpbcs/2022.13.4.16>

Pérez, C., Navarro, A., Saldaña, M. T., Masramón, X., Pérez, M., & Rejas, J. (2013). Clinical and resource utilization patterns in patients with refractory neuropathic pain prescribed pregabalin for the first time in routine medical practice in primary care settings in Spain. *Pain Med*, *14*(12), 1954-1963. <https://doi.org/10.1111/pme.12276>

Ramírez-Maestre, C., Reyes-Pérez, Á., Esteve, R., López-Martínez, A. E., Bernardes, S., & Jensen, M. P. (2020). Opioid Pain Medication Prescription for Chronic Pain in Primary Care Centers: The Roles of Pain Acceptance, Pain Intensity, Depressive Symptoms, Pain Catastrophizing, Sex, and Age. *Int J Environ Res Public Health*, *17*(17). <https://doi.org/10.3390/ijerph17176428>

Robinson, R. L., Schnitzer, T. J., Barlow, S., Berry, M., Bushmakin, A. G., Cappelleri, J. C., Tive, L., Jackson, J., Jackson, J., & Viktrup, L. (2022). Satisfaction with Medications Prescribed for Osteoarthritis: A Cross-Sectional Survey of Patients and Their Physicians in the United States. *Pain Ther*, *11*(1), 191-208. <https://doi.org/10.1007/s40122-021-00350-0>

Sicras-Mainar, A., Tornero-Tornero, C., Vargas-Negrín, F., Lizarraga, I., & Rejas-Gutierrez, J. (2020). Health outcomes and costs in patients with osteoarthritis and chronic pain treated with opioids in Spain: the OPIOIDS real-world study. *Ther Adv Musculoskelet Dis*, *12*, 1759720x20942000. <https://doi.org/10.1177/1759720x20942000>

Taguchi, T., Nozawa, K., Parsons, B., Yoshiyama, T., Ebata, N., Igarashi, A., & Fujii, K. (2019). Effectiveness of pregabalin for treatment of chronic cervical radiculopathy with upper limb radiating pain: an 8-week, multicenter prospective observational study in Japanese primary care settings. *J Pain Res*, *12*, 1411-1424. <https://doi.org/10.2147/jpr.S191906>

Vogler, C. N., Sattovia, S., Salazar, L. Y., Leung, T. I., & Botchway, A. (2017). Assessing outcomes of educational videos in group visits for patients with chronic pain at an academic primary care clinic. *Postgrad Med*, *129*(5), 524-530. <https://doi.org/10.1080/00325481.2017.1324228>

Wayne, P. M., Buring, J. E., Eisenberg, D. M., Osypiuk, K., Gow, B. J., Davis, R. B., Witt, C. M., & Reinhold, T. (2019). Cost-Effectiveness of a Team-Based Integrative Medicine Approach to the Treatment of Back Pain. *J Altern Complement Med*, *25*(S1), S138-s146. <https://doi.org/10.1089/acm.2018.0503>

White, L. D., Summers, P., & Scott, A. (2018). Changes in Clinical Status after Completion of an Interdisciplinary Pain Management Programme Incorporating Pain Neurophysiology Education. *Physiother Can*, *70*(4), 382-392. <https://doi.org/10.3138/ptc.2016-72.ep>

Zinboonyahgoon, N., Saengsomsuan, N., Chaikittiporn, N., Wangnamthip, S., Kositamongkol, C., & Phisalprapa, P. (2023). Cost-Utility and Cost-Effectiveness Analysis of Spinal Cord Stimulation for Chronic Refractory Pain in the Context of Developing Country. *Pain Physician*, *26*(1), 69-79.
