# Supplementary material for: Systematic Review of Outcome Measures in Pharmacologically Managed Chronic Pain: Informing a New Outcome Framework for Healthcare Provider‐Led Pharmacotherapy Services
Source: J Eval Clin Pract. 2025 Feb 26;31(2):e70029. doi: 10.1111/jep.70029 (PMC11865632; doi:10.1111/jep.70029)
Supplement: Supplementary file 4 — Supporting information. [file JEP-31-0-s005.docx]

**Table S4:** Baseline characteristics of included population / outcome measures and follow up

| Study ID | Baseline characteristics of included studies | | | | | | | | Outcome measures and follow up | | | |
| --- | --- | --- | --- | --- | --- | --- | --- | --- | --- | --- | --- | --- |
|  | **Study Groups** | **Setting** | **Age Mean(SD)** | **Gender Male(%)** | **Type of Pain** | **Purpose of Treatment** | **Drug Treatment** | **Pain Care Provider** | **Baseline Measure Conducted (Yes/No)** | **Timing of Measure After Intervention** | **Performer of the Measure** | **Follow-up Duration** |
| (Nadkarni et al., 2022) | "•G1: monotherapy •G2: combination therapy" | Pain Super Specialty Outpatient Department at KIMS Hospital and Research Centre | 44.68(12.07) | 69 (69) | neuropathic pain | Treating ongoing pain | NSAIDs, weak opioids, strong opioids, TCAs, Anticonvulsants | Pain specialist | yes | 1st week and 6th week | patient | 6 weeks |
| (Robinson et al., 2022) | G1: patients. G2: physicians | US primary care | G1: 64.9 (11.3) | G1: 226(39.5) | Osteoarthritis | Treating ongoing pain | such as acetaminophen, oral and topical non- steroidal anti-inflammatory drugs [NSAIDs], duloxetine, and opioids including tramadol | physician | yes | 1 week | patient/ physcian | 4 months |
| (Zinboonyahgoon et al., 2023) | all one group | The pain clinic at Siriraj Hospital, a tertiary care center in Thailand | average (range): 47.4 (15-79) years | 16(54%) | Chronic Refractory Pain | Treating ongoing pain | conventional management (CMM) | physician | yes | 6 months/ 1y / 2y/ 3y | patient: pain/QoL. Cost-effectiveness: societal | 36 months |
| (Moreira de Barros et al., 2021) | "•G1: 175 methadone •G2: 87 morphine" | outpatient specialized Pain PAIN Reports® Management and Palliative Care Unit of the Teaching Hospital of Botucatu Medical School—UNESP | "•G1: 62.1 (15.5) •G2: 55.5 (12.02)" | "•G1: 59 (33.7%) •G2: 38 (44.3%)" | chronic pain (Nocioceptive , neuropathic and mixed type ) | Treating ongoing pain | Methadone, Morphine | physician | yes | every 40 days throw out the year | patient | 1 year |
| (Ganguly et al., 2021) | G1: Non-white. G2: White. | HIV primary care | N/A | 108 (65.1 %) | pain in HIV patients | Treating ongoing pain | opioids | physician | yes | at least 1 visit during the study and after the baseline | patient | 18 months |
| (Gudin et al., 2020) | "G1 Topical patch G2:Control group" | outpatient setting | 46 | 72(36) | "Arthritis Neuropathy/radiculopathy Myofascial/musculoskeletal" | Treating ongoing pain | OTC and Prescribed NSAID, Opioid, anticonvulsant, Muscle relaxant | Clinicians | yes | Days 3, 7, and 14 | Patient | 28 days |
| (Kaboré et al., 2020) | G1: improvers. G2: non-improvers | Quebec Pain Registry (QPR) - pain clinic | 51.3 (12.5) | 49 (42.2) | chronic noncancer pain (mainly neuropathic pain ) | Treating ongoing pain | opioids | primary care physicians | yes | 6, 12, 24 months | "patient self- administered questionnaire and a nurse-administered questionnaire." | 24 months |
| (Ramírez-Maestre et al., 2020) | G1: Participants Prescribed Opioids. G2: Participants not Prescribed Opioids | primary care centers | 45.4 (12.9) | 274 (41) | chronic back pain | Treating ongoing pain | "opioids and other analgesics " | primary care physicians | yes | at least 3 months from baseline | patient | 42 months |
| (Sicras-Mainar et al., 2020) | G1: Knee/hip. G2: spine. G3: others. | primary care centers | 70.8 (14.3) | 10791(28) | osteoarthritis | Treating ongoing pain | opioids and other analgesics | physician | yes | 12, 24, and 36months | patient | 36 months |
| (Lee et al., 2020) | G1: low pain relief. G2: high pain relief | Texas A&M Health Science Center Opioid Task Force | N/A | N/A | chronic noncancer pain | Treating ongoing pain | narcotics, non-narcotics or opioids | primary care physicians | no | at least after 6 months of analgesics use | patient | 2 months |
| (Taguchi et al., 2019) | "•G1: pregabalin •G2: usual care" | Japanese primary care | "•G1: 58.3(15.9) •G2: 66.4(15.8)" | "•G1: 77 (53.1) •G2: 68 (30.4)" | chronic cervical radiculopathy with upper limb radiating pain | Treating ongoing pain | pregabalin, anticonvulsant,Antidepressant, Opioid and non-opioid Analgesics | Primary care physicians | yes | week 4 & week 8 | "Physician / at w8 (PGIC) &(CGIC): patient or clinician" | 8 weeks |
| (Wayne et al., 2019) | G1: Osher Clinical Center. G2: Non-Osher Clinical Center | Osher Clinical Center (OCC) based at a tertiary academic hospital | G1: 50.18 (16.65). G2: 52.08 (15.88) | G1: 42 (31.3). G2: 38 (26.4) | Back Pain | Treating ongoing pain | Integrative or conventional care | physician | yes | 3, 6, 12 months | patient: pain/QoL. Cost-effectiveness: societal | 12 months |
| (McCann et al., 2018) | G1: Remained on Opioids. G2: Who Weaned Off | Rural Primary Care Office | "•G1: 65.88 •G2: 68.25" | "•G1: 12 (71%) •G2: 8 (67%)" | nonmalignant chronic pain (NMCP) ( neck, Upper and lower back pain, shoulder, knee, polyarthralgia, peripheral neuropathy) | continuing or weaning opioids | opioids | Primary care physicians | yes | several visits | patient / The physician aided in the completion of the forms, if incomplete, in the context of the visit | 18 weeks |
| (Elsesser & Cegla, 2017) | G1: patients with continuous opioid treatment. G2: patients receiving nonopioid analgesics | Clinic for Pain Medicine in primary care | G1: 62.74 (15.34). G2: 65.86 (13.48) | G1: 24%. G2: 26.5% | Non cancer chronic pain including neuropathic pain | Treating ongoing pain | opioids, different kinds of analgesics or coanalgesics | physician | yes | after 3 months | patients | 3 months |
| (Ghodke et al., 2018) | G1: short acting opioids. G2: long acting opioids. | UNC Internal Medicine Pain Service (IMPS) | G1: 59.6 (10.32). G2: 59.9 (8.83). | 54 male (G1: 33 (28.0). G2: 21 (39.6)). | moderate to severe osteoarthritis | Treating ongoing pain | opioids | physician | yes | 1 - 2 weeks after baseline | patient | 1 - 2 weeks |
| (Vogler et al., 2017) | all one group | primary care | 58 (±11) | 23% | chronic non-cancer pain (Back pain, upper and lower exremity pain, neck pain, neuropathy, arthritis, fibromyalgia ) | Treating ongoing pain | opioid | physician | yes | 20 visits | Patient | 18 months |
| (White et al., 2018) | G1: patient completed the program | rehabilitation centre | 45.9 (11.7) | 57 (55.9) | myofascial pain, other musculoskeletal pain, neuropathic pain, mixed pain, psychological diagnoses (pain disorder), and complex regional pain syndrome. | Treating ongoing pain | opioid | case coordinator, psychologist, occupational therapist, physical therapist, kinesiologist, pharmacist, and physician | yes | 3 weeks | Patient | 6 weeks |
| (Igarashi et al., 2015) | G1: Pregabalin. G2: usual care | primary care | Mean age: 60 years | N/A | chronic low back pain with accompanying neuropathic pain (CLBP-NeP | Treating ongoing pain | pregabalin | physician | yes | monthly | patient: pain/QoL. Cost-effectiveness: payer and societal | 12 months |
| (Dunn et al., 2014) | methadone-maintaned patients | "Addiction Treatment Services methadone-maintenance clinic" | mean (SEM) 46.00 ± 0.87 | 64 (47) | "Upper and lower extremities back,head, neck, shoulders ,chest, abdomen and hips pain " | "treatment of opioid use disorder" | opioid , Ibuprofen, Gabapentin and non pharmacological approaches | Physician Led- Addiction Treatment Services | No | Approx. 8 months | self-report questionnaire | N/A |
| (Jouini et al., 2014) | all one group, patients with NCCP | primary care | 58.4 (12.5) | 157(32.3) | Osteoarthritis and other osteopathologie,Chronic back pain,Chronic neck pain,Fibromyalgia, Tendinitis, bursitis, capsulitis, epicondylitis | Treating ongoing pain | acetaminophen, nonsteroidal anti-inflammatory drugs, anticonvulsants, antidepressants, muscle relaxants, and opioids | primary-care physician / pharmacists | no | at least after 6 months of analgesics use | patient | 6 months |
| (Ashworth et al., 2013) | G1: no opioids. G2: low dose opioid. G3: medium dose opioid. G4: high dose opioid. | UK primary care | G1: 45.77 (9.87). G2: 46.27 (9.36). G3: 44.07 (10.69). G4: 47.64 (9.39). | G1: 44%. (100% = G2: 33% + G3: 27% + G4: 24% ) | low back pain | Treating ongoing pain | opioids | physician | yes | after 6 months | patient | 6 months |
| (Pérez et al., 2013) | "•G1: pregabalin as monotherapy •G2: added pregabalin to their existing pain treatments •G3: other pain treatments were prescribed" | routine medical prac- tice in primary care | "•G1: 58.6 (12.5) •G2: 59.7 (13.0) •G3: 60.4 (12.4)" | "•G1: 289 (39.6) •G2: 307 (42.2) •G3: 84 (44.7)" | refractory chronic neuropathic pain | Treating ongoing pain | pregabalin and analgesics | physician | yes | after 12 weeks | patient | 12 weeks |
| (Blanco Tarrio et al., 2013) | G1: Patients treated with pregabalin either as monotherapy or in combination | spanish Primary care | 58.5 (13.7) | 689(41) | neuropathic pain | Treating ongoing pain | Non-pharmacological therapy as (Physiotherapy and Acupuncture) and pharmacological therapy as (pregabalin, anticonvulsant,Antidepressant, Opioid and non-opioid Analgesics ) | Primary care physicians | yes | after 3 months | patient | 3 months |

Ashworth, J., Green, D. J., Dunn, K. M., & Jordan, K. P. (2013). Opioid use among low back pain patients in primary care: Is opioid prescription associated with disability at 6-month follow-up? *Pain*, *154*(7), 1038-1044. <https://doi.org/10.1016/j.pain.2013.03.011>

Blanco Tarrio, E., Gálvez Mateos, R., Zamorano Bayarri, E., López Gómez, V., & Pérez Páramo, M. (2013). Effectiveness of pregabalin as monotherapy or combination therapy for neuropathic pain in patients unresponsive to previous treatments in a Spanish primary care setting. *Clin Drug Investig*, *33*(9), 633-645. <https://doi.org/10.1007/s40261-013-0116-7>

Dunn, K. E., Brooner, R. K., & Clark, M. R. (2014). Severity and interference of chronic pain in methadone-maintained outpatients. *Pain Med*, *15*(9), 1540-1548. <https://doi.org/10.1111/pme.12430>

Elsesser, K., & Cegla, T. (2017). Long-term treatment in chronic noncancer pain: Results of an observational study comparing opioid and nonopioid therapy. *Scand J Pain*, *17*, 87-98. <https://doi.org/10.1016/j.sjpain.2017.07.005>

Ganguly, A. P., Lira, M. C., Lodi, S., Forman, L. S., Colasanti, J. A., Williams, E. C., Liebschutz, J. M., Del Rio, C., Samet, J. H., & Tsui, J. I. (2021). Race and satisfaction with pain management among patients with HIV receiving long-term opioid therapy. *Drug Alcohol Depend*, *222*, 108662. <https://doi.org/10.1016/j.drugalcdep.2021.108662>

Ghodke, A., Barquero, S., Chelminski, P. R., & Ives, T. J. (2018). Short-Acting Opioids Are Associated with Comparable Analgesia to Long-Acting Opioids in Patients with Chronic Osteoarthritis with a Reduced Opioid Equivalence Dosing. *Pain Med*, *19*(11), 2191-2195. <https://doi.org/10.1093/pm/pnx245>

Gudin, J. A., Dietze, D. T., & Hurwitz, P. L. (2020). Improvement of Pain and Function After Use of a Topical Pain Relieving Patch: Results of the RELIEF Study. *J Pain Res*, *13*, 1557-1568. <https://doi.org/10.2147/jpr.S258883>

Igarashi, A., Akazawa, M., Murata, T., Taguchi, T., Sadosky, A., Ebata, N., Willke, R., Fujii, K., Doherty, J., & Kobayashi, M. (2015). Cost-effectiveness analysis of pregabalin for treatment of chronic low back pain in patients with accompanying lower limb pain (neuropathic component) in Japan. *Clinicoecon Outcomes Res*, *7*, 505-520. <https://doi.org/10.2147/ceor.S89833>

Jouini, G., Choinière, M., Martin, E., Perreault, S., Berbiche, D., Lussier, D., Hudon, E., & Lalonde, L. (2014). Pharmacotherapeutic management of chronic noncancer pain in primary care: lessons for pharmacists. *J Pain Res*, *7*, 163-173. <https://doi.org/10.2147/jpr.S56884>

Kaboré, J. L., Saïdi, H., Dassieu, L., Choinière, M., & Pagé, M. G. (2020). Predictors of Long-Term Opioid Effectiveness in Patients With Chronic Non-Cancer Pain Attending Multidisciplinary Pain Treatment Clinics: A Quebec Pain Registry Study. *Pain Pract*, *20*(6), 588-599. <https://doi.org/10.1111/papr.12883>

Lee, S., Smith, M. L., Dahlke, D. V., Pardo, N., & Ory, M. G. (2020). A Cross-Sectional Examination of Patients' Perspectives About Their Pain, Pain Management, and Satisfaction with Pain Treatment. *Pain Med*, *21*(2), e164-e171. <https://doi.org/10.1093/pm/pnz244>

McCann, K. S., Barker, S., Cousins, R., Franks, A., McDaniel, C., Petrany, S., & Riley, E. (2018). Structured Management of Chronic Nonmalignant Pain with Opioids in a Rural Primary Care Office. *J Am Board Fam Med*, *31*(1), 57-63. <https://doi.org/10.3122/jabfm.2018.01.170163>

Moreira de Barros, G. A., Baradelli, R., Rodrigues, D. G., Toffoletto, O., Domingues, F. S., Gayoso, M. V., Lopes, A., Barros Afiune, J., & Nunes Guimarães, G. M. (2021). Use of methadone as an alternative to morphine for chronic pain management: a noninferiority retrospective observational study. *Pain Rep*, *6*(4), e979. <https://doi.org/10.1097/pr9.0000000000000979>

Nadkarni, S., Ramesh, J., Bk, A., & Girish, K. (2022). A Prospective Observational Study On Pattern Of Drug Use For Neuropathic Pain In A Tertiary Hospital. *Research Journal of Pharmaceutical, Biological and Chemical Sciences*, *13*, 101-109. <https://doi.org/10.33887/rjpbcs/2022.13.4.16>

Pérez, C., Navarro, A., Saldaña, M. T., Masramón, X., Pérez, M., & Rejas, J. (2013). Clinical and resource utilization patterns in patients with refractory neuropathic pain prescribed pregabalin for the first time in routine medical practice in primary care settings in Spain. *Pain Med*, *14*(12), 1954-1963. <https://doi.org/10.1111/pme.12276>

Ramírez-Maestre, C., Reyes-Pérez, Á., Esteve, R., López-Martínez, A. E., Bernardes, S., & Jensen, M. P. (2020). Opioid Pain Medication Prescription for Chronic Pain in Primary Care Centers: The Roles of Pain Acceptance, Pain Intensity, Depressive Symptoms, Pain Catastrophizing, Sex, and Age. *Int J Environ Res Public Health*, *17*(17). <https://doi.org/10.3390/ijerph17176428>

Robinson, R. L., Schnitzer, T. J., Barlow, S., Berry, M., Bushmakin, A. G., Cappelleri, J. C., Tive, L., Jackson, J., Jackson, J., & Viktrup, L. (2022). Satisfaction with Medications Prescribed for Osteoarthritis: A Cross-Sectional Survey of Patients and Their Physicians in the United States. *Pain Ther*, *11*(1), 191-208. <https://doi.org/10.1007/s40122-021-00350-0>

Sicras-Mainar, A., Tornero-Tornero, C., Vargas-Negrín, F., Lizarraga, I., & Rejas-Gutierrez, J. (2020). Health outcomes and costs in patients with osteoarthritis and chronic pain treated with opioids in Spain: the OPIOIDS real-world study. *Ther Adv Musculoskelet Dis*, *12*, 1759720x20942000. <https://doi.org/10.1177/1759720x20942000>

Taguchi, T., Nozawa, K., Parsons, B., Yoshiyama, T., Ebata, N., Igarashi, A., & Fujii, K. (2019). Effectiveness of pregabalin for treatment of chronic cervical radiculopathy with upper limb radiating pain: an 8-week, multicenter prospective observational study in Japanese primary care settings. *J Pain Res*, *12*, 1411-1424. <https://doi.org/10.2147/jpr.S191906>

Vogler, C. N., Sattovia, S., Salazar, L. Y., Leung, T. I., & Botchway, A. (2017). Assessing outcomes of educational videos in group visits for patients with chronic pain at an academic primary care clinic. *Postgrad Med*, *129*(5), 524-530. <https://doi.org/10.1080/00325481.2017.1324228>

Wayne, P. M., Buring, J. E., Eisenberg, D. M., Osypiuk, K., Gow, B. J., Davis, R. B., Witt, C. M., & Reinhold, T. (2019). Cost-Effectiveness of a Team-Based Integrative Medicine Approach to the Treatment of Back Pain. *J Altern Complement Med*, *25*(S1), S138-s146. <https://doi.org/10.1089/acm.2018.0503>

White, L. D., Summers, P., & Scott, A. (2018). Changes in Clinical Status after Completion of an Interdisciplinary Pain Management Programme Incorporating Pain Neurophysiology Education. *Physiother Can*, *70*(4), 382-392. <https://doi.org/10.3138/ptc.2016-72.ep>

Zinboonyahgoon, N., Saengsomsuan, N., Chaikittiporn, N., Wangnamthip, S., Kositamongkol, C., & Phisalprapa, P. (2023). Cost-Utility and Cost-Effectiveness Analysis of Spinal Cord Stimulation for Chronic Refractory Pain in the Context of Developing Country. *Pain Physician*, *26*(1), 69-79.
