# Supplementary material for: Systematic Review of Outcome Measures in Pharmacologically Managed Chronic Pain: Informing a New Outcome Framework for Healthcare Provider‐Led Pharmacotherapy Services
Source: J Eval Clin Pract. 2025 Feb 26;31(2):e70029. doi: 10.1111/jep.70029 (PMC11865632; doi:10.1111/jep.70029)
Supplement: Supplementary file 5 — Supporting information. [file JEP-31-0-s004.docx]

**Table S5:** Summary of outcome measures used

| Study ID | Key Clinical Measurements | | | | | | | | | | | | | | | | | | | Tool validated? | |
| --- | --- | --- | --- | --- | --- | --- | --- | --- | --- | --- | --- | --- | --- | --- | --- | --- | --- | --- | --- | --- | --- |
|  | **Patient Experience** | | **Medication related adverse events** | | | **Cost Effectiveness** | | | | | | **Medication Optimization** | | | **Health related Quality of life** | | | | |  |  |
|  | **Domain** | **Measurement Tool** | **Domain** | **Measurement Tool** | | **Domain** | | **Model used** | **Measurement Tool** | | | **Domain** | **Measurement Tool** | | **Domain** | | | **Measurement Tool** | |  |  |
| (Nadkarni et al., 2022) | N/A | N/A | tolerability | patients reporting any side effects or by the history of drug related adverse events | | N/A | | N/A | N/A | | | N/A | N/A | | Pain Intensity | | | Short-Form McGill Pain Questionnaire | | not mentioned | |
| (Robinson et al., 2022) | Expectations of the effectiveness of medications prescribed | 5-point Likert scale | N/A | N/A | | N/A | | N/A | N/A | | | N/A | N/A | | Pain Intensity | | | 11-point numeric rating scale | | not mentioned | |
|  |  |  |  |  |  |  |  |  |  |  |  |  |  |  | functional limitations due to OA | | | Physical Function subscale | |  |  |
|  |  |  |  |  |  |  |  |  |  |  |  |  |  |  | (mobility, self-care, usual activities, pain/discomfort, and anxiety/depression | | | EQ-5D-5L | |  |  |
| (Zinboonyahgoon et al., 2023) | N/A | N/A | N/A | N/A | | (ICUR)  and  (ICER) | | Decision tree and Markov model | ICUR was calculated per QALY gained, utilizing EQ-5D-5L health questionnaires.  *ICER was calculated per Numeric Rating Pain Score (NRS) reduction. | | | N/A | N/A | | quality of life | | | EQ-5D-5L health questionnaires | | yes (for all measures) | |
| (Moreira de Barros et al., 2021) | N/A | N/A | occurrence of side effects | registered in the medical records | | N/A | | N/A | N/A | | | N/A | N/A | | Pain Intensity | | | 11-point numeric verbal scale | | yes (for all measures) | |
|  |  |  |  |  |  |  |  |  |  |  |  |  |  |  | performance status | | | Karnofsky Performance Status | |  |  |
| (Moreira de Barros et al., 2021) | patient satisfaction with the clinic, medications, physician interaction, and nursing interaction  patient-related barriers towards pain management | patient satisfaction with pain management Questions  modified version of the Barriers Questionnaire (BQ-II) | N/A | N/A | | N/A | | N/A | N/A | | | N/A | N/A | | pain intensity | | | Brief Pain Inventory | | not mentioned | |
| (Gudin et al., 2020) | Satisfaction | satisfaction ratings scale | Rate of side effect | Patient reported side effect | | N/A | | N/A | N/A | | | Changes in Concurrent Pain Medications | Survey | | pain severity and interference | | | brief pain inventory (BPI) | | not mentioned | |
| (Kaboré et al., 2020) | N/A | N/A | N/A | N/A | | N/A | | N/A | N/A | | | N/A | N/A | | pain, enjoyment of life, and general activity | | | "PEG (pain, enjoyment of life, and general activity) Scale" | | yes (for all measures) | |
|  |  |  |  |  |  |  |  |  |  |  |  |  |  |  | Pain Intensity | | | Brief Pain Inventory (BPI) | |  |  |
|  |  |  |  |  |  |  |  |  |  |  |  |  |  |  | "mental health–related quality of life (mQoL) and physical health–related quality of life (pQoL)" | | | SF-12v2 Health Survey | |  |  |
|  |  |  |  |  |  |  |  |  |  |  |  |  |  |  | Pain Catastrophizing | | | Pain Catastrophizing Scale | |  |  |
|  |  |  |  |  |  |  |  |  |  |  |  |  |  |  | Depression and anxiety | | | Beck Depression Inventory-I (BDI) | |  |  |
|  |  |  |  |  |  |  |  |  |  |  |  |  |  |  | "the presence of neuropathic pain qualities through self- report and physical examination" | | | DN4 Questionnaire | |  |  |
|  |  |  |  |  |  |  |  |  |  |  |  |  |  |  | "pain history (eg, pain duration and frequency)" | | | Nurse-Administered Questionnaire | |  |  |
| (Ramírez-Maestre et al., 2020)  (Ramírez-Maestre et al., 2020) | N/A | N/A | N/A | N/A | | N/A | | N/A | N/A | | | N/A | N/A | | Depression and anxiety | | | Hospital Anxiety and Depression Scale | | not mentioned | |
|  |  |  |  |  |  |  |  |  |  |  |  |  |  |  | Pain Catastrophizing | | | Pain Catastrophizing Scale (PCS) | |  |  |
|  |  |  |  |  |  |  |  |  |  |  |  |  |  |  | Pain Acceptance | | | Chronic Pain Acceptance Questionnaire (CPAQ-SV | |  |  |
|  |  |  |  |  |  |  |  |  |  |  |  |  |  |  | Pain Intensity | | | No specific name (scale ranging from 0 to 10, with 0 indicating “No pain” and 10 indicating “Pain as intense as you could imagine.”) | |  |  |
| (Ramírez-Maestre et al., 2020) | N/A | N/A | N/A | N/A | N/A | | N/A | | | N/A | N/A | | | N/A | | Pain Intensity | 11-points numeric rating scale (NRS) | | not mentioned | |  |
|  |  |  |  |  |  |  |  |  |  |  |  |  |  |  | | basic activities of daily living | Barthel index | |  |  |  |
|  |  |  |  |  |  |  |  |  |  |  |  |  |  |  |  | mental health | Mini-Mental State Examination (MMSE) | |  |  |  |
| (Lee et al., 2020) | confidence in their regular primary care phys ician’s knowledge about pain management and independent variables (rate the extent to which they were allowed to participate in decisions about their pain treatment) | American Pain Society Patient Outcome Questionnaire | N/A | N/A | | N/A | | N/A | N/A | | | N/A | N/A | | pain relief, patient beliefs | | | American Pain Society Patient Outcome Questionnaire | | not mentioned | |
| (Taguchi et al., 2019)  (Wayne et al., 2019)  (Taguchi et al., 2019) | perceived change in health status from the perspective of the patient or clinician | The Patient Global Impression of Change (PGIC) and Clinical Global Impression of Change (CGIC) | tolerability & SE | AE recorded (Unknown) | | N/A | | N/A | N/A | | | N/A | N/A | | pain, personal care, work, sleep | | | Neck Disability Index (NDI) | | not mentioned | |
|  |  |  |  |  |  |  |  |  |  |  |  |  |  |  | mobility, self-care, usual activities, pain/discomfort, anxiety/depression | | | EuroQol five dimensions five level (EQ-5D-5L) | |  |  |
|  |  |  |  |  |  |  |  |  |  |  |  |  |  |  |  | | |  | |  |  |
|  |  |  |  |  |  |  |  |  |  |  |  |  |  |  | sleep disturbance | | | Pain-Related Sleep-Interference Scale (PRSIS) | |  |  |
| (Wayne et al., 2019) | N/A | N/A | N/A | N/A | | Incremental cost-effectiveness ratio (ICER) | | N/A | QALYs based on the SF-12 assessments | | | N/A | N/A | | quality of life | | | SF-12 quality-of-life (QOL) | | not mentioned | |
|  |  |  |  |  |  |  |  |  |  |  |  |  |  |  | Back pain-related functional limitation | | | modified Roland Disability Questionnaire (RDQ) | |  |  |
| (Taguchi et al., 2019) | N/A | N/A | risks | SOAPP-R diversion risk assessment tool | | N/A | | N/A | N/A | | | N/A | N/A | | pain’s intensity and effect on quality of life | | | Brief Pain Inventory Short Form | | yes (for all measures) | |
|  |  |  | side effects | Flesch-Kincaid15 system | |  |  |  |  |  |  |  |  |  | depression | | | Zung Depression scale | |  |  |
|  |  |  |  |  |  |  |  |  |  |  |  |  |  |  | low back pain | | | Roland disability rating scale | |  |  |
| (Elsesser & Cegla, 2017) | N/A | N/A | N/A | N/A | | N/A | | N/A | N/A | | | N/A | N/A | | Pain severity and functioning | | | German Pain Questionnaire (GPQ; German: Deutscher Schmerzfragebogen) | | not mentioned | |
|  |  |  |  |  |  |  |  |  |  |  |  |  |  |  | satisfaction with pain | | | numeric rating scale (NRS) | |  |  |
|  |  |  |  |  |  |  |  |  |  |  |  |  |  |  | Disability | | | The modified Pain Disability Index (mPDI) | |  |  |
|  |  |  |  |  |  |  |  |  |  |  |  |  |  |  | intensity, persistence and disability/interference | | | Chronic Pain Grade Scale (CPG) | |  |  |
|  |  |  |  |  |  |  |  |  |  |  |  |  |  |  | Neuropathic pain | | | painDETECT questionnaire | |  |  |
|  |  |  |  |  |  |  |  |  |  |  |  |  |  |  | QoL | | | The WHOQoL-BREF | |  |  |
|  |  |  |  |  |  |  |  |  |  |  |  |  |  |  | General wellbeing | | | Marburg Questionnaire on Habit- ual Health (MQHH; original German version: Fragebogen zum Habituellen Wohlbefinden) | |  |  |
|  |  |  |  |  |  |  |  |  |  |  |  |  |  |  | Depression and anxiety | | | Hospital Anxiety and Depression Scale (HADS) | |  |  |
| (Ghodke et al., 2018) | N/A | N/A | N/A | N/A | | N/A | | N/A | N/A | | | N/A | N/A | | Pain Intensity | | | Brief Pain Inventory (BPI) | | not mentioned | |
| (Vogler et al., 2017) | knowledge assessment | . Pre- and post-knowledge assessment questions | N/A | N/A | | N/A | | N/A | N/A | | | Change in morphine equivalent dose | "chart review and the Illinois Prescription Monitoring Program" | | functional status | | | "Pain Intensity, Enjoyment of life, and General Activity (PEG) score" | |  | |
|  | patient satisfaction | Patient Satisfaction Survey |  |  |  |  |  |  |  |  |  |  |  |  | functional status | | | e Oswestry Disability Index Survey | |  |  |
| (White et al., 2018) | N/A | N/A | N/A | N/A | | N/A | | N/A | N/A | | | Morphine intake | morphine equivalent dose (MEQ) recorded by the pharmacist for each patient | | depression | | | Beck Depression Inven- tory [BDI] | | yes (for all measures) | |
|  |  |  |  |  |  |  |  |  |  |  |  |  |  |  | pain severity / pain interference | | | Brief Pain Inventory [BPI] | |  |  |
|  |  |  |  |  |  |  |  |  |  |  |  |  |  |  | pain knowledge | | | Neurophysiology of Pain Question- naire—Revised [NPQ–R] | |  |  |
|  |  |  |  |  |  |  |  |  |  |  |  |  |  |  | pain intensity | | | numer- ical pain rating scale (NPRS | |  |  |
| (Igarashi et al., 2015) | N/A | N/A | N/A | N/A | | incremental cost-effectiveness ratio (ICER) | | State-transition model - Markov model | QALYs from EQ-5D-5L questionnaire and Numerical Rating Scale (NRS) | | | N/A | N/A | | Pain Intensity | | | numerical rating scale (NRS) | | not mentioned | |
|  |  |  |  |  |  |  |  |  |  |  |  |  |  |  | quality of life | | | five-level, five-dimension EuroQol health status measure (EQ-5D-5L) | |  |  |
|  |  |  |  |  |  |  |  |  |  |  |  |  |  |  | sleep | | | (pain-related interference with sleep) | |  |  |
| (Dunn et al., 2014) | N/A | N/A | N/A | N/A | | N/A | | N/A | N/A | | | N/A | N/A | | Pain intensity | | | brief pain inventory (BPI) | | yes (for all measures) | |
| (Jouini et al., 2014) | patient Experience | The Pain Treatment Satisfaction Scale (PTSS) | side effects | self-administered questionnaire: | | N/A | | N/A | N/A | | | N/A | N/A | | pain intensity | | | numerical intensity-scale | | yes (for all measures) | |
|  |  |  |  |  |  |  |  |  |  |  |  |  |  |  | pain interferes with their general activity, walking ability, mood, normal work, relations with other people, sleep, enjoyment of life, self-care, recreational activities, and social activities. | | | modified Brief Pain Inventory (BPI) | |  |  |
|  |  |  |  |  |  |  |  |  |  |  |  |  |  |  | Impact of pain on sleep | | | Chronic Pain Sleep Inventory | |  |  |
|  |  |  |  |  |  |  |  |  |  |  |  |  |  |  | Depression and anxiety | | | Hospital Anxiety and Depression Scale | |  |  |
|  |  |  |  |  |  |  |  |  |  |  |  |  |  |  | physiological effects, fatalism, communication, harmful effects | | | The Barriers Questionnaire II | |  |  |
| (Ashworth et al., 2013) | N/A | N/A | N/A | N/A | | N/A | | N/A | N/A | | | N/A | N/A | | Disability | | | (Roland-Morris Disability Ques- tionnaire [RMDQ]) | | not mentioned | |
|  |  |  |  |  |  |  |  |  |  |  |  |  |  |  | pain intensity | | | numerical rating scale | |  |  |
|  |  |  |  |  |  |  |  |  |  |  |  |  |  |  | Anxiety and Depression | | | Hospital Anxiety and Depression Scale (HADS) | |  |  |
|  |  |  |  |  |  |  |  |  |  |  |  |  |  |  | Fear of movemen | | | Tampa Scale of Kinesiophobia | |  |  |
|  |  |  |  |  |  |  |  |  |  |  |  |  |  |  | assess patients’ beliefs about their ability to accomplish activities despite pain | | | Pain Self-Efficacy Questionnaire | |  |  |
| (Pérez et al., 2013) | N/A | N/A | N/A | N/A | | N/A | | N/A | LWDE | | | N/A | N/A | | 1. work, social life, and family life/home responsibilities. | | | 1. Sheehan Disability Inventory (SDI). | | yes (for all measures) | |
|  |  |  |  |  |  |  |  |  |  |  |  |  |  |  | 2. self-perceived aspects of sleep: sleep disturbances, snoring, and awakening short of breath or with a headache, sleep adequacy, daytime somnolence, and quantity of sleep. | | | 2. Medical Outcomes Study Sleep Scale (MOS-Sleep). | |  |  |
|  |  |  |  |  |  |  |  |  |  |  |  |  |  |  | 3. Hospital Anxiety and Depression Scales. | | | 3. Hospital Anxiety and Depression Scales. | |  |  |
|  |  |  |  |  |  |  |  |  |  |  |  |  |  |  | 4.describe patient health in five dimensions (mobility, self-care, usual activities, pain/ discomfort, and anxiety/depression. | | | 4. EuroQoL (EQ-5D) questionnaire. | |  |  |
|  |  |  |  |  |  |  |  |  |  |  |  |  |  |  | 5. pain | | | 5. Short Form McGill Pain Questionnaire | |  |  |
| (Blanco Tarrio et al., 2013) | Overall satisfaction | Treatment Satisfaction for Medication Questionnaire (SATMED-Q) | N/A | N/A | | N/A | | N/A | N/A | | | N/A | N/A | | pain descriptors | | | DN4 Questionnaire | | yes (for all measures) | |
|  |  |  |  |  |  |  |  |  |  |  |  |  |  |  | intensity of pain and its impact on activities of daily living | | | the Brief Pain Inventory (BPI) | |  |  |

Ashworth, J., Green, D. J., Dunn, K. M., & Jordan, K. P. (2013). Opioid use among low back pain patients in primary care: Is opioid prescription associated with disability at 6-month follow-up? *Pain*, *154*(7), 1038-1044. <https://doi.org/10.1016/j.pain.2013.03.011>

Blanco Tarrio, E., Gálvez Mateos, R., Zamorano Bayarri, E., López Gómez, V., & Pérez Páramo, M. (2013). Effectiveness of pregabalin as monotherapy or combination therapy for neuropathic pain in patients unresponsive to previous treatments in a Spanish primary care setting. *Clin Drug Investig*, *33*(9), 633-645. <https://doi.org/10.1007/s40261-013-0116-7>

Dunn, K. E., Brooner, R. K., & Clark, M. R. (2014). Severity and interference of chronic pain in methadone-maintained outpatients. *Pain Med*, *15*(9), 1540-1548. <https://doi.org/10.1111/pme.12430>

Elsesser, K., & Cegla, T. (2017). Long-term treatment in chronic noncancer pain: Results of an observational study comparing opioid and nonopioid therapy. *Scand J Pain*, *17*, 87-98. <https://doi.org/10.1016/j.sjpain.2017.07.005>

Ghodke, A., Barquero, S., Chelminski, P. R., & Ives, T. J. (2018). Short-Acting Opioids Are Associated with Comparable Analgesia to Long-Acting Opioids in Patients with Chronic Osteoarthritis with a Reduced Opioid Equivalence Dosing. *Pain Med*, *19*(11), 2191-2195. <https://doi.org/10.1093/pm/pnx245>

Gudin, J. A., Dietze, D. T., & Hurwitz, P. L. (2020). Improvement of Pain and Function After Use of a Topical Pain Relieving Patch: Results of the RELIEF Study. *J Pain Res*, *13*, 1557-1568. <https://doi.org/10.2147/jpr.S258883>

Igarashi, A., Akazawa, M., Murata, T., Taguchi, T., Sadosky, A., Ebata, N., Willke, R., Fujii, K., Doherty, J., & Kobayashi, M. (2015). Cost-effectiveness analysis of pregabalin for treatment of chronic low back pain in patients with accompanying lower limb pain (neuropathic component) in Japan. *Clinicoecon Outcomes Res*, *7*, 505-520. <https://doi.org/10.2147/ceor.S89833>

Jouini, G., Choinière, M., Martin, E., Perreault, S., Berbiche, D., Lussier, D., Hudon, E., & Lalonde, L. (2014). Pharmacotherapeutic management of chronic noncancer pain in primary care: lessons for pharmacists. *J Pain Res*, *7*, 163-173. <https://doi.org/10.2147/jpr.S56884>

Kaboré, J. L., Saïdi, H., Dassieu, L., Choinière, M., & Pagé, M. G. (2020). Predictors of Long-Term Opioid Effectiveness in Patients With Chronic Non-Cancer Pain Attending Multidisciplinary Pain Treatment Clinics: A Quebec Pain Registry Study. *Pain Pract*, *20*(6), 588-599. <https://doi.org/10.1111/papr.12883>

Lee, S., Smith, M. L., Dahlke, D. V., Pardo, N., & Ory, M. G. (2020). A Cross-Sectional Examination of Patients' Perspectives About Their Pain, Pain Management, and Satisfaction with Pain Treatment. *Pain Med*, *21*(2), e164-e171. <https://doi.org/10.1093/pm/pnz244>

Moreira de Barros, G. A., Baradelli, R., Rodrigues, D. G., Toffoletto, O., Domingues, F. S., Gayoso, M. V., Lopes, A., Barros Afiune, J., & Nunes Guimarães, G. M. (2021). Use of methadone as an alternative to morphine for chronic pain management: a noninferiority retrospective observational study. *Pain Rep*, *6*(4), e979. <https://doi.org/10.1097/pr9.0000000000000979>

Nadkarni, S., Ramesh, J., Bk, A., & Girish, K. (2022). A Prospective Observational Study On Pattern Of Drug Use For Neuropathic Pain In A Tertiary Hospital. *Research Journal of Pharmaceutical, Biological and Chemical Sciences*, *13*, 101-109. <https://doi.org/10.33887/rjpbcs/2022.13.4.16>

Pérez, C., Navarro, A., Saldaña, M. T., Masramón, X., Pérez, M., & Rejas, J. (2013). Clinical and resource utilization patterns in patients with refractory neuropathic pain prescribed pregabalin for the first time in routine medical practice in primary care settings in Spain. *Pain Med*, *14*(12), 1954-1963. <https://doi.org/10.1111/pme.12276>

Ramírez-Maestre, C., Reyes-Pérez, Á., Esteve, R., López-Martínez, A. E., Bernardes, S., & Jensen, M. P. (2020). Opioid Pain Medication Prescription for Chronic Pain in Primary Care Centers: The Roles of Pain Acceptance, Pain Intensity, Depressive Symptoms, Pain Catastrophizing, Sex, and Age. *Int J Environ Res Public Health*, *17*(17). <https://doi.org/10.3390/ijerph17176428>

Robinson, R. L., Schnitzer, T. J., Barlow, S., Berry, M., Bushmakin, A. G., Cappelleri, J. C., Tive, L., Jackson, J., Jackson, J., & Viktrup, L. (2022). Satisfaction with Medications Prescribed for Osteoarthritis: A Cross-Sectional Survey of Patients and Their Physicians in the United States. *Pain Ther*, *11*(1), 191-208. <https://doi.org/10.1007/s40122-021-00350-0>

Taguchi, T., Nozawa, K., Parsons, B., Yoshiyama, T., Ebata, N., Igarashi, A., & Fujii, K. (2019). Effectiveness of pregabalin for treatment of chronic cervical radiculopathy with upper limb radiating pain: an 8-week, multicenter prospective observational study in Japanese primary care settings. *J Pain Res*, *12*, 1411-1424. <https://doi.org/10.2147/jpr.S191906>

Vogler, C. N., Sattovia, S., Salazar, L. Y., Leung, T. I., & Botchway, A. (2017). Assessing outcomes of educational videos in group visits for patients with chronic pain at an academic primary care clinic. *Postgrad Med*, *129*(5), 524-530. <https://doi.org/10.1080/00325481.2017.1324228>

Wayne, P. M., Buring, J. E., Eisenberg, D. M., Osypiuk, K., Gow, B. J., Davis, R. B., Witt, C. M., & Reinhold, T. (2019). Cost-Effectiveness of a Team-Based Integrative Medicine Approach to the Treatment of Back Pain. *J Altern Complement Med*, *25*(S1), S138-s146. <https://doi.org/10.1089/acm.2018.0503>

White, L. D., Summers, P., & Scott, A. (2018). Changes in Clinical Status after Completion of an Interdisciplinary Pain Management Programme Incorporating Pain Neurophysiology Education. *Physiother Can*, *70*(4), 382-392. <https://doi.org/10.3138/ptc.2016-72.ep>

Zinboonyahgoon, N., Saengsomsuan, N., Chaikittiporn, N., Wangnamthip, S., Kositamongkol, C., & Phisalprapa, P. (2023). Cost-Utility and Cost-Effectiveness Analysis of Spinal Cord Stimulation for Chronic Refractory Pain in the Context of Developing Country. *Pain Physician*, *26*(1), 69-79.
